# Supplementary material for: Proteomic and genetic predictors and risk scores of cardiovascular diseases in persons living with HIV
Source: Front Cardiovasc Med. 2026 Jan 28;12:1726272. doi: 10.3389/fcvm.2025.1726272 (PMC12892344; doi:10.3389/fcvm.2025.1726272)
Supplement: Supplementary file 1 [file Datasheet1.pdf]

A. More on Methods

How were incident CVD cases identified and validated to represent true events?

In each of the trials, CVD events were reported as part of ongoing clinical data throughout the follow-up. Events were reported with supporting documentation. A blinded endpoint review committee (ERC) for each trial compared the submitted documentation against pre-existing diagnostic criteria. Events adjudicated by the ERC as confirmed or probable were considered as true events.

Baseline CVD

In START a CVD event within 6 months before randomization was exclusionary because CVD was part of the primary outcome for the trial. No such limitations on prior CVD for ESPRIT, SMART, and FIRST.

Incidence rates for CVD in the trials

Study	No. in Trial	Median Follow-up	Pooled CVD rate during follow-up
FIRST	1397	4.8 years	0.56 per 100 py
START	4684	5.8 years	0.16 per 100 py
SMART	5472	2.4 years	0.95 per 100 py
ESPRIT	4111	6.9 years	0.46 per 100 py

Note: For START, the study used a data cut through 28 Apr 2018. For the other trials, follow-up cited in the manuscript is through all follow-up.

Proteomics Data from Olink, Normalization and Quality Control

The data are median intensity normalized, meaning that the data are adjusted to make the median value for each assay on each plate equal to the median for that of the other plates. The data are presented as normalized protein expression values (NPX) and are reported on the $\log_2$ scale. Four internal controls for each sample are added to monitor the quality of assay performance and the quality of each individual sample. There are two steps of quality control: 1) each sample plate with above 0.2 NPX when evaluated on the standard deviation of the internal controls pass this first step and 2) samples that deviate less than 0.3 NPX from the median value of the controls for each individual sample pass this step. We used 5 Olink multiplex panels (Cardiovascular II, Cardiovascular III, Immune Response, Inflammation, and Cardiometabolic) with each panel having 92 proteins. The identity of the 92 proteins for the Olink panels used can be found here https://olink.com/products/olink-target-96.

Integrative Analysis of Genetics and Proteomics Data

We considered a multivariate approach to investigate the associations between the genetics and proteomics data, and to determine genetic variants and proteins that are correlated and discriminate between CVD cases and controls. Proteins that appeared on multiple panels were all considered in the integrative analysis. We used the sparse integrative analysis (SIDA) approach[11] for this purpose. SIDA is a joint association and classification method that allows to simultaneously model correlations between two or more data types (in our case the genetics

and proteomics data) while allowing for discrimination between two or more groups (in our case CVD cases and controls). SIDA allows the detection of key variables that both maximize associations between the genetics and proteomics data and discriminate between CVD cases and controls. For statistical rigor, we coupled SIDA with resampling techniques to determine key molecules. We obtained 100 bootstrap training and testing datasets. On each bootstrap data, we fitted a logistic regression model of our outcome with each protein and genetic variant. The genetic variant was coded as the number of minor alleles. Molecules which were statistically significant (p-value < 0.05) were used in the SIDA algorithm to integrate the genetic and proteomics data. By embedding the univariate step into the integration step, we ensure that variables that went into SIDA discriminated between CVD cases and controls. Proteins and genes passing the univariate filtering step and were frequently selected by SIDA to discriminate between cases and controls were chosen as candidate variables for downstream analyses.

More on Pathway Analysis of Molecules Identified in Proteo-genomic Integrative Analysis
Regarding upstream regulators, tumor necrosis factor (TNF), lipopolysaccharide (LPS) and tetradecanoylphorbol acetate were each predicted to be activated. In particular, the cytokine TNF was predicted to be activated with a z-score of 2.077 and overlap p-value 5.65E-06. Six (HGF, LTBR, IL6, UPAR, CCL11, GT) out of 8 genes known in the literature to be upregulated by TNF were also upregulated in our dataset, which is consistent with activation of TNF. LPS was predicted to be activated with a z-score of 2.385 and overlap p-value 2.68E-05 with 6 (HGF, IL6, UPAR, CCL11, CCL18, PLA2G7) out of 8 genes consistent with activation of LPS. Tetradecanoylphorbol acetate was predicted to be activated (z-score 2.156, overlap p-value 3.27E-05) with 5 (HGF, IL6, UPAR, CCL11, CCL18) out of 6 genes consistent with activation of tetradecanoylphorbol acetate.

B. Description of proteins used in protein score

Inflammation Panel:

Hepatocyte Growth Factor (HGF):

The protein HGF and its receptor c-MET are involved in tissue repair and respond to tissue injury. HGF has been proposed as a potential clinical biomarker for CVD[45] HGF has already been shown to be associated with stroke, CHD, atherosclerosis, and the progression of atherosclerosis in an ethnically diverse, general population[29,30]

Fibroblast growth factor 19 (FGF19):

FGF19, a member of the fibroblast growth factor (FGF), is a protein that in humans is encoded by the gene FG19. Proteins in the FGF family have been implicated in a variety of biological processes that includes cell growth, tissue repair, tumor growth and invasion[48], glucose and lipid metabolism[49]. They have been suggested as therapeutic biomarkers for chronic diseases such as obesity, type 2 diabetes, cancer, and kidney and cardiovascular disease in the general population[49].

Immune Response Panel:

C-C motif chemokine 11 (CCL11):

CCL-11 belongs to the family of cytokines implicated in immunoregulatory and inflammatory processes. Increased levels of CCL11 have been associated with coronary artery disease[38]. Higher levels of CCL11 have been linked to CD4+ T-cells loss[39].

CLEC6A
C-type lectin domain containing 6A (CLEC6A) is a protein that is encoded in humans by the CLEC6A gene. A recent Mendelian randomization analysis in PLWH that used the same cohort we used identified CLEC6A as potentially causally related to CVD[32].

Interleukin-6 (IL6):

IL6 has been extensively studied in both healthy and HIV positive populations. IL6 is a marker of inflammation and coagulation. Increased levels of plasma IL6 has been shown to associated with increased risk of CVD, atherosclerosis, and mortality in an HIV positive population even when treated with ART[25–27].

Cardiovascular 2 Panel:

Gastrotropin (GT):

Gastrotropin, also known as the ileal fatty acid binding protein, (FABP6) is a member of the fatty acid-binding protein (FABPs) family, which regulates general metabolic function via FABPs central role in fatty acid transport, metabolism, and storage. FABPs have been associated with a number of diseases including cardiovascular disease and are thought to serve an integral role in metabolic function[34]. FABP6 is more specifically known to be involved in bile acid metabolism. There has been shown to be a protective association between FABP6 Thr79Met polymorphism and incident type 2 diabetes[50].

A disintegrin and metalloproteinase with thrombospondin motifs 13 (ADAMTS13)

A disintegrin and metalloproteinase with thrombospondin motifs 13 is a protein that in human is encoded by the gene ADAMTS13. Low levels of the protein ADAMTS13 is suggested to be associated with in an increased risk of ischemic stroke, myocardial infarction and cerebrovascular disease in the general population[51], and is thought to contribute to increased cardiovascular risk in persons with HIV[52]. In an HIV study, ADAMTS13 antigen and

Interleukin-1 receptor-like 2 (IL1RL2)

IL1RL2 is a protein that in humans is encoded by the IL1RL2 gene. It is a member of the interlukin 1 receptor family, which is implicated in inflammatory diseases[53]. IL1 is suggested to contribute to the initiation, formation, growth and rupture of atherosclerosis plaques[54].

Cardiometabolic Panel

Platelet-activating factor acetylhydrolase (PLA2G7):

PLA2G7 is found in both high-density lipoprotein (HDL) and low-density lipoprotein (LDL). In population studies it has been shown that overexpression of PLA2G7 is associated with increased coronary heart disease (CHD)[28,31]. It is thought that with individuals with low LDL cholesterol levels it can help predict CHD risk[28,31].

C-C motif chemokine 18 (CCL18):

CCL-18 belongs to the family of cytokines implicated in immunoregulatory and inflammatory processes. CCL18 upregulation has been reported in a number of diseases, including HIV infection, atherosclerosis and pulmonary fibrosis[36,37].

Cardiovascular 3 Panel

Lymphotoxin beta receptor (**LTBR**)
LTBR, also known as the tumor tumor necrosis factor receptor superfamily member 3 (TNFRSF3) is a member of the tumor necrosis factor receptor family and is implicated in apoptosis and cytokine release. This protein was found to be associated with the presence of plaque in PLWH[55].

urokinase-type plasminogen activator receptor or urokinase receptor (uPAR)
uPAR is a protein encoded in humans by the plasminogen activator, urokinase receptor gene (PLAUR). It is a member of the plasminogen activator system which has been implicated in biological processes such as hemostatis and inflammation[56,57]. UPAR is predicted to be upregulated during conditions of injury and inflammation[58]. Research suggests that in persons with HIV, uPAR expression is upregulated with activation of monocytes and T lymphocytes[59].

Secretoglobin Family 3A Member (SCGB3A2):

A case-control study in a Korean population on SNPs in the SCGB3A2 gene potentially contributes to susceptibility to asthma[35]. The SCGB3A2 gene is also known as the uterus globulin associated protein 1 (UGRP1) found that UGRP1 may be able to predict graves' disease patients who develop hypothyroidism[60].

insulin like growth factor binding protein (IGFBP7)

IGFBP7 (insulin like growth factor binding protein 7) is a protein which in humans is encoded by the gene IGFBP7. Studies have reported the association of IGFBP7 in a variety of cancers. Recent work suggest IGFBP7 as a marker of cellular senescence, insulin resistance and atherosclerosis[61–63].

Table S1: Log-odds ratios of proteins used in Ingenuity Pathway Analysis

Protein	Log-odds ratios	Odds Ratios	P-value
IL6	0.420019	1.521991	0.000195

CCL11	0.342293	1.408173	0.002502
CLEC6A	0.342984	1.409146	0.003178
HGF	0.575619	1.778232	7.63E-06
FGF19	0.269885	1.309813	0.015346
ADAMTS13	-0.36212	0.696197	0.063394
IL1RL2	-0.24638	0.781623	0.045265
GAST	0.224594	1.251814	0.047525
CCL18	0.33387	1.396362	0.003123
PLA2G7	0.274521	1.315901	0.013146
LTBR	0.453132	1.573232	0.000197
PLAUR	0.360565	1.43414	0.001672
SCGB3A2	0.338422	1.402732	0.002127
IGFBP7	0.488998	1.630682	6.49E-05

Table S2. Coefficients from Baseline + Protein Score Model

**Variable**	**OR**	**LCI**	**UCI**	**p-value**
Gender	0.565	0.223	1.319	0.204
Age	0.986	0.957	1.014	0.324
CVD	5.647	1.574	27.06	0.014
CD4	1	0.999	1.001	0.773
Lipid-lowering medication	1.217	0.629	2.318	0.553
BP lowering medication	1.711	0.835	3.483	0.139
Diabetes	0.733	0.251	2.021	0.555
Black Race	1.277	0.601	2.662	0.517
Protein Score	2.356	1.776	3.19	< 0.001

Table S3: Logistic Regression Model of CVD on Each of the Standardized Proteins Used to Develop the Protein Score (n=360)

	OR	SE	p-value	LCI	UCI
Baseline + CCL11	1.36	0.127	0.015	1.064	1.751
Baseline + CLEC6A	1.419	0.13	0.007	1.106	1.84
Baseline + IL6	1.439	0.125	0.004	1.129	1.847
Baseline + HGF	1.623	0.137	< 0.001	1.252	2.141
Baseline + FGF19	1.378	0.122	0.009	1.088	1.757
Baseline + ADAMTS13	0.63	0.221	0.037	0.403	0.944
Baseline + IL1RL2	0.761	0.141	0.053	0.572	0.993
Baseline + GT	0.747	0.132	0.027	0.572	0.959
Baseline + CCL18	1.455	0.123	0.002	1.15	1.866
Baseline + PLA2G7	1.356	0.127	0.017	1.058	1.746
Baseline + LTBR	1.514	0.129	0.001	1.185	1.971
Baseline + UPAR	1.373	0.123	0.01	1.085	1.759
Baseline + SCGB3A2	1.422	0.12	0.003	1.127	1.803
Baseline + IGFBP7	1.509	0.137	0.003	1.163	1.992

Table S4: Incremental Contribution of Individual Proteins and SNPs and Protein Score to CVD Risk When Added to Baseline Model (n=360)

Model	AUC	LCB	UCB	NRI Cases	NRI Controls	Overall NRI
Baseline Model	0.612	0.5495	0.6746			
Baseline + Individual Proteins						
Baseline + CCL11	0.647	0.585	0.708	0.0847	0.048	0.1328
Baseline + CLEC6A	0.653	0.5916	0.7135	0.0847	0.1703	0.2551
Baseline + IL6	0.679	0.6202	0.7377	0.0339	0.1965	0.2304
Baseline + HGF	0.671	0.6121	0.7306	0.136	0.162	0.297
Baseline + FGF19	0.653	0.5911	0.7147	0.0339	0.1441	0.178
Baseline + ADAMTS13	0.637	0.5763	0.6978	0.0847	0.1092	0.1939
Baseline + IL1RL2	0.645	0.5842	0.7059	0.0678	0.214	0.2818
Baseline + GT	0.636	0.5745	0.697	0.271	0.048	0.319
Baseline + CCL18	0.653	0.5924	0.7139	0	0.188	0.188
Baseline + PLA2G7	0.649	0.5871	0.7108	0.0678	0.2227	0.2905
Baseline + LTBR	0.663	0.6009	0.7258	0.0508	0.1965	0.2474
Baseline + UPAR	0.661	0.6009	0.7209	0.153	0.258	0.41
Baseline + SCGB3A2	0.656	0.5948	0.717	0.0339	0.2664	0.3003
Baseline + IGFBP7	0.646	0.5826	0.7086	-0.0508	0.1703	0.1195
**Baseline + Protein Score**	0.742	0.6865	0.7968	0.305	0.345	0.65
Baseline + Individual SNPs						
Baseline + 4 PCs + rs11895665	0.649	0.588	0.711	-0.017	0.223	0.206
Baseline + 4 PCs + rs2240688	0.684	0.624	0.745	0.254	0.162	0.416

Baseline + 4 PCs + rs4696483	0.667	0.607	0.728	-0.169	0.467	0.298
Baseline + 4 PCs + rs34308112	0.667	0.607	0.732	-0.203	0.616	0.412
Baseline + 4 PCs + rs10456432	0.694	0.636	0.751	0.492	-0.013	0.478
Baseline + 4 PCs + rs3808528	0.650	0.590	0.710	0.220	0.135	0.356
Baseline + 4 PCs + rs17252559	0.660	0.600	0.721	-0.169	0.450	0.280
Baseline + 4 PCs + rs3816208	0.653	0.592	0.715	-0.186	0.406	0.220
Baseline + 4 PCs + rs9410490	0.660	0.599	0.721	0.271	0.118	0.389
Baseline + 4 PCs + rs16940029	0.635	0.573	0.697	0.305	-0.066	0.240
Baseline + 4 PCs + rs115708227	0.670	0.612	0.729	0.746	-0.345	0.401
Baseline + 4 PCs + rs17053844	0.636	0.574	0.698	-0.051	0.266	0.216
Baseline + 4 PCs + rs80067004	0.646	0.584	0.708	-0.085	0.284	0.199
Baseline + 4 PCs + rs73320494	0.663	0.604	0.722	0.525	-0.109	0.416
Baseline + 4 PCs + rs12602462	0.654	0.592	0.716	0.254	0.013	0.267
**Baseline + SNP Score + 4 PCs**	0.829	0.784	0.874	0.492	0.467	0.959
**Baseline + Protein Score + SNP Score + 4 PCs**	0.858	0.819	0.897	0.525	0.546	1.071

Table S5. Coefficients from Baseline + Protein Score Model (ART Subset)

Variable	OR	LCI	UCI	p-value
Gender	0.69	0.20	2.02	0.51
Age	0.99	0.95	1.02	0.43
CVD at Baseline	2.67	0.61	14.28	0.21
CD4	1.00	1.00	1.00	0.83
Lipid-lowering medication	1.30	0.60	2.74	0.50
BP lowering medication	2.14	0.84	5.44	0.11

Diabetes	0.64	0.15	2.45	0.53
Black Race	0.99	0.36	2.53	0.98
Protein Score	2.27	1.65	3.20	< 0.001

Table S6. Selected Coefficients from Baseline + SNP Score + 4 PCs Model

**Variable**	**OR**	**LCI**	**UCI**	**p-value**
Gender	0.698	0.262	1.722	0.45
Age	0.99	0.959	1.022	0.551
CVD	9.11	2.101	50.901	0.006
CD4	0.999	0.998	1.001	0.41
Lipid-lowering medication	1.349	0.646	2.782	0.42
BP lowering medication	1.202	0.544	2.637	0.646
Diabetes	1.082	0.351	3.192	0.888
Black Race	0.624	0.039	22.791	0.766
SNP Score	4.586	3.214	6.803	< 0.001

Table S7: Logistic Regression Model of CVD on Each of the Standardized SNPs Used to Develop the Genetic Score (n=360). The ORs for SNPs are obtained from fitting a multivariable logistic regression of CVD with each standardized SNP, adjusting for covariates. SNPs are treated as continuous variables.

	OR	SE	p-value	LCI	UCI
Baseline + 4 PCs + X2.238552973_T	1.335	0.127	0.023	1.04	1.717
Baseline + 4 PCs + X4.15970349_G	0.671	0.136	0.003	0.51	0.871
Baseline + 4 PCs + X4.154619255_T	1.481	0.133	0.003	1.142	1.93
Baseline + 4 PCs + X5.53444623_A	1.654	0.126	< 0.001	1.298	2.133
Baseline + 4 PCs + X6.35671651_C	0.552	0.147	< 0.001	0.408	0.729
Baseline + 4 PCs + X8.23158775_A	1.305	0.122	0.029	1.028	1.661
Baseline + 4 PCs + X8.53603859_G	1.367	0.121	0.01	1.078	1.736
Baseline + 4 PCs + X8.97614625_A	1.391	0.119	0.006	1.102	1.762
Baseline + 4 PCs + X9.92089100_C	0.703	0.132	0.007	0.539	0.905
Baseline + 4 PCs + X12.109617728_G	1.318	0.145	0.057	0.993	1.758
Baseline + 4 PCs + X12.109644408_A	0.623	0.158	0.003	0.443	0.829
Baseline + 4 PCs + X13.53625421_T	1.248	0.118	0.061	0.989	1.579
Baseline + 4 PCs + X15.101934803_G	1.342	0.123	0.017	1.053	1.712
Baseline + 4 PCs + X17.45373571_C	0.632	0.148	0.002	0.467	0.836
Baseline + 4 PCs + X17.78146016_T	1.29	0.123	0.038	1.014	1.645

Table S8. Selected Coefficients from Baseline + SNP Score + 4 PCs Model (ART Subset)

Variable	OR	LCI	UCI	p-value
Gender	0.98	0.28	3.01	0.97
Age	0.99	0.96	1.03	0.71

CVD at Baseline	5.42	1.03	33.49	0.05
CD4	1.00	1.00	1.00	0.29
Lipid-lowering medication	1.45	0.62	3.34	0.38
BP lowering medication	1.48	0.52	4.22	0.46
Diabetes	1.08	0.24	4.50	0.92
Black Race	0.99	0.03	122.53	> 0.99
SNP Score	3.82	2.63	5.81	< 0.001

Table S9. Selected Coefficients from Baseline + Protein Score + SNP Score + 4 PCs Model (ART Subset)

**Variable**	**OR**	**LCI**	**UCI**	**p-value**
Gender	1.22	0.32	4.12	0.75
Age	0.99	0.95	1.02	0.45
CVD at Baseline	4.28	0.80	26.59	0.10
CD4	1.00	1.00	1.00	0.62
Lipid-lowering medication	1.53	0.62	3.77	0.35
BP lowering medication	2.00	0.69	6.10	0.22
Diabetes	0.70	0.13	3.38	0.67
Black Race	0.25	0.0057	48.30	0.52
Protein Score	2.24	1.55	3.35	< 0.001
SNP Score	3.90	2.61	6.10	< 0.001

We conduct additional analyses where we do not include CVD at baseline in the models.

Table S10: Coefficients from Baseline + Protein Score Model (no CVD at Baseline Subset)

**Variable**	**OR**	**95% CI**	**p-value**
Gender	0.57	(0.22, 1.34)	0.21
Age	0.98	(0.96, 1.01)	0.31
CD4	1.00	(1.00, 1.00)	0.77
Lipid-lowering medication	1.42	(0.72, 2.79)	0.31
BP lowering medication	1.42	(0.67, 2.98)	0.36

Diabetes	0.79	(0.25, 2.30)	0.67
Black Race	1.36	(0.63, 2.88)	0.43
Protein Score	2.41	(1.81, 3.28)	<0.001

Table S11: Selected Coefficients from Baseline + SNP Score + 4 PCs Model (no CVD at Baseline Subset)

Variable	OR	95% CI	p-value
Gender	0.68	(0.26, 1.68)	0.42
Age	0.99	(0.96, 1.02)	0.48
CD4	1.00	(1.00, 1.00)	0.44
Lipid-lowering medication	1.40	(0.66, 2.92)	0.37
BP lowering medication	1.13	(0.49, 2.54)	0.78
Diabetes	1.17	(0.36, 3.57)	0.79
Black Race	0.75	(0.05, 28.0)	0.85
SNP Score	4.24	(2.99, 6.25)	<0.001

Table S12 Selected Coefficients from Baseline + Protein Score + SNP Score + 4 PCs Model (no CVD at Baseline Subset)

Variable	OR	95% CI	p-value
Gender	0.82	(0.29, 2.15)	0.70
Age	0.98	(0.95, 1.01)	0.29
CD4	1.00	(1.00, 1.00)	0.78
Lipid-lowering medication	1.49	(0.67, 3.27)	0.32
BP lowering medication	1.16	(0.48, 2.76)	0.74
Diabetes	0.85	(0.24, 2.81)	0.79
Black Race	0.30	(0.02, 12.0)	0.44

Protein Score	2.28	(1.64, 3.27)	<0.001
SNP Score	4.30	(2.96, 6.53)	<0.001

Figures

Figure S1 SNPs QC for Inflammation pathway

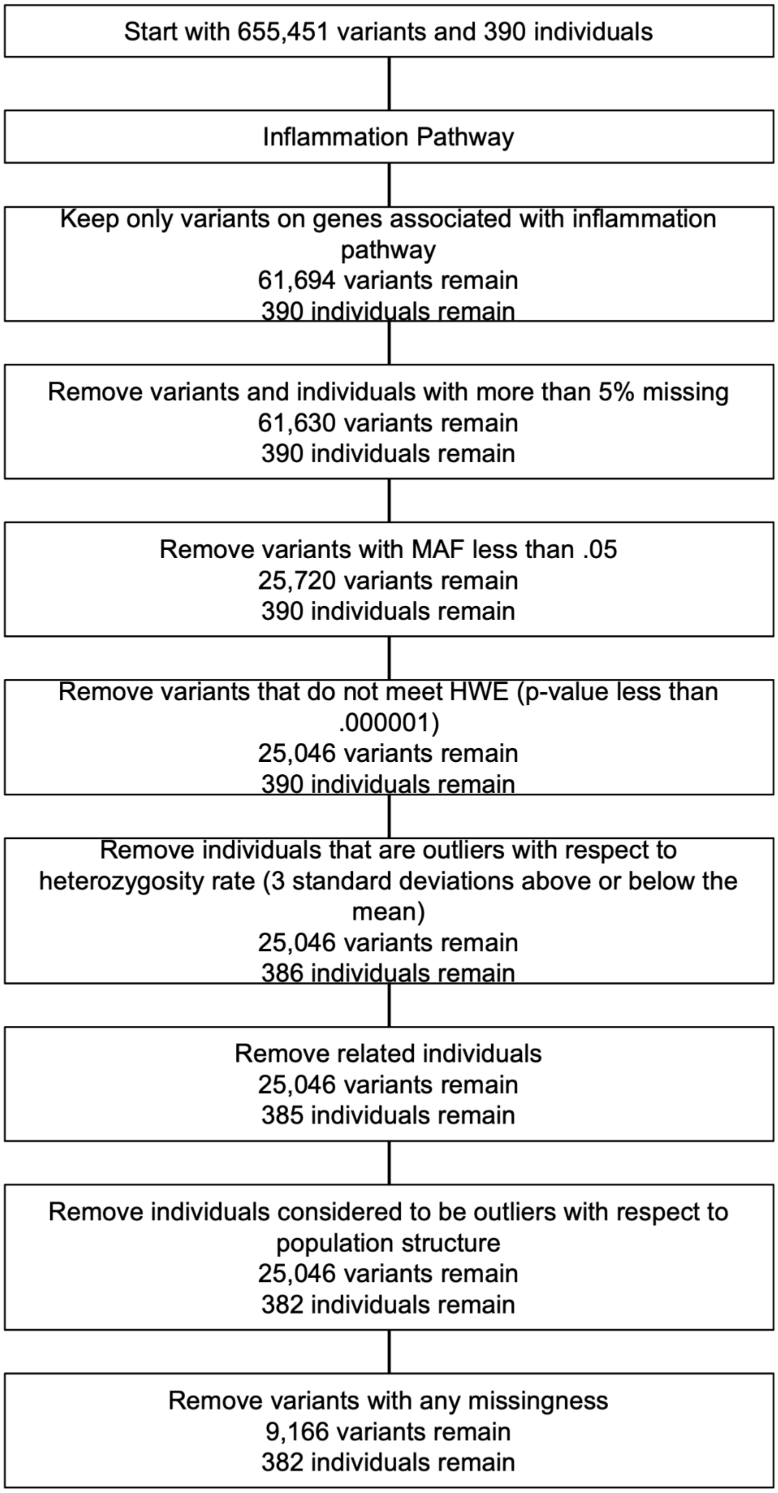

Figure S2 Pipeline for our framework

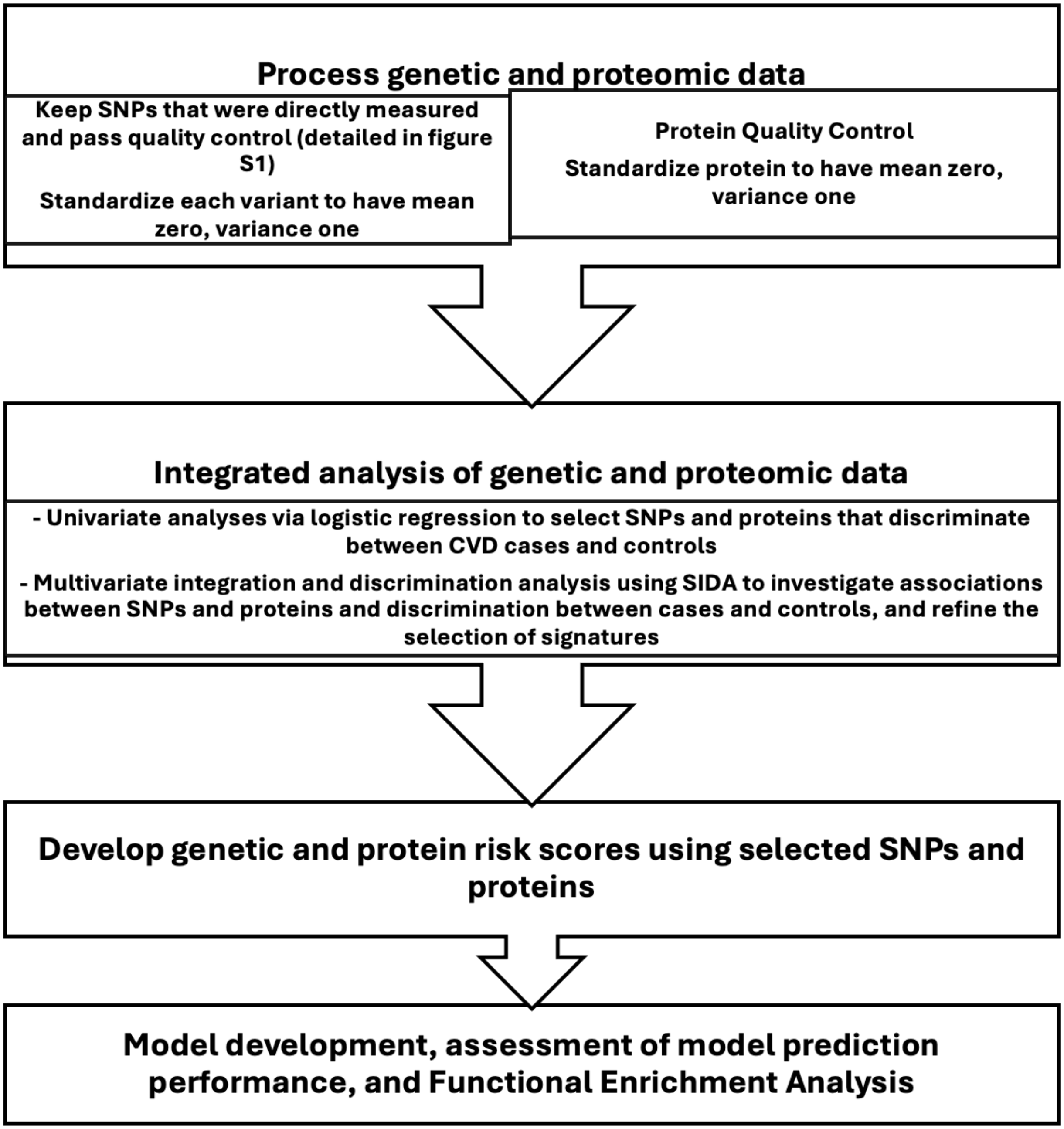

Figure S3 Distribution of ancestry-adjusted genetic and proteomics score across demographic variables.

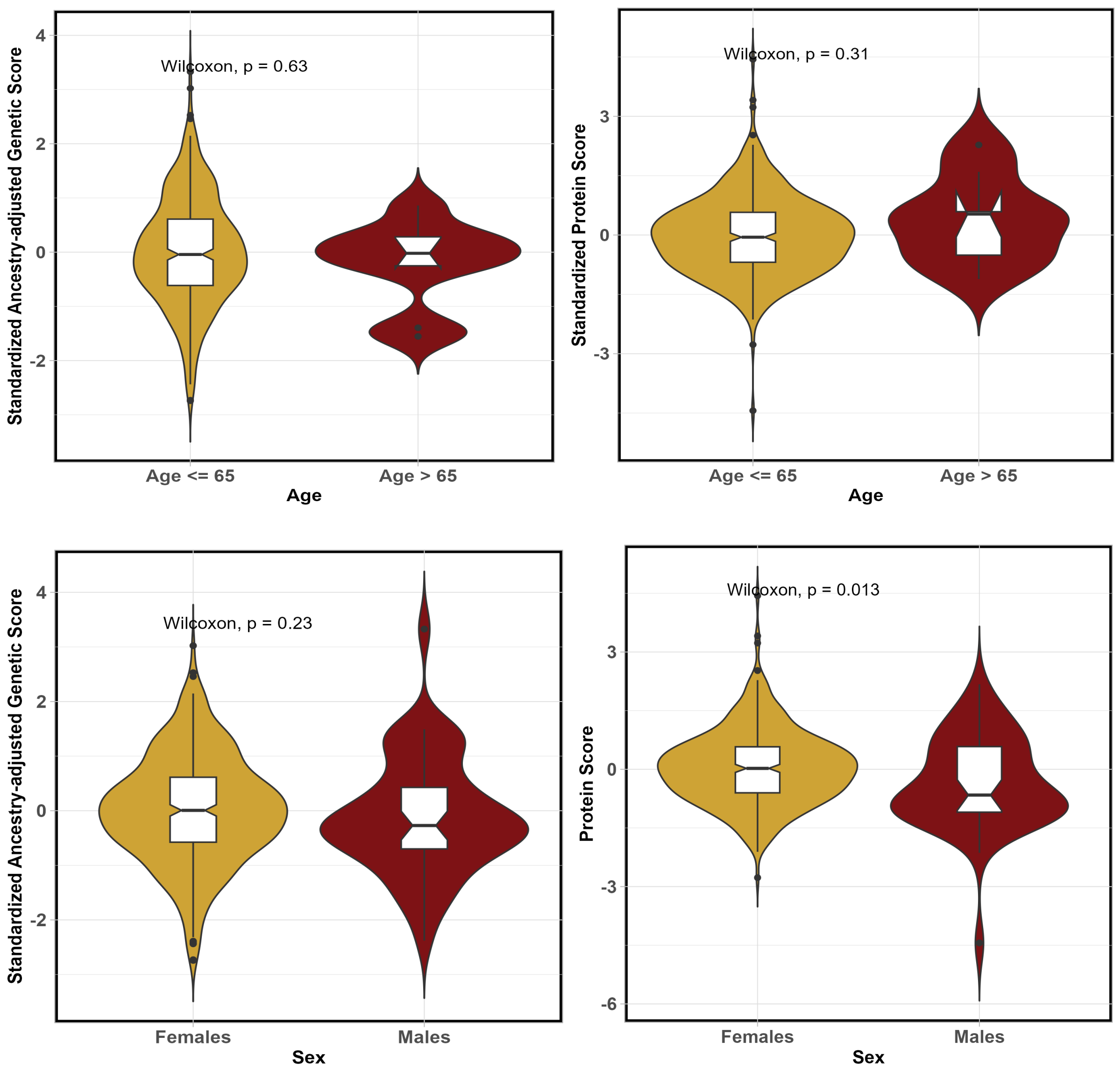

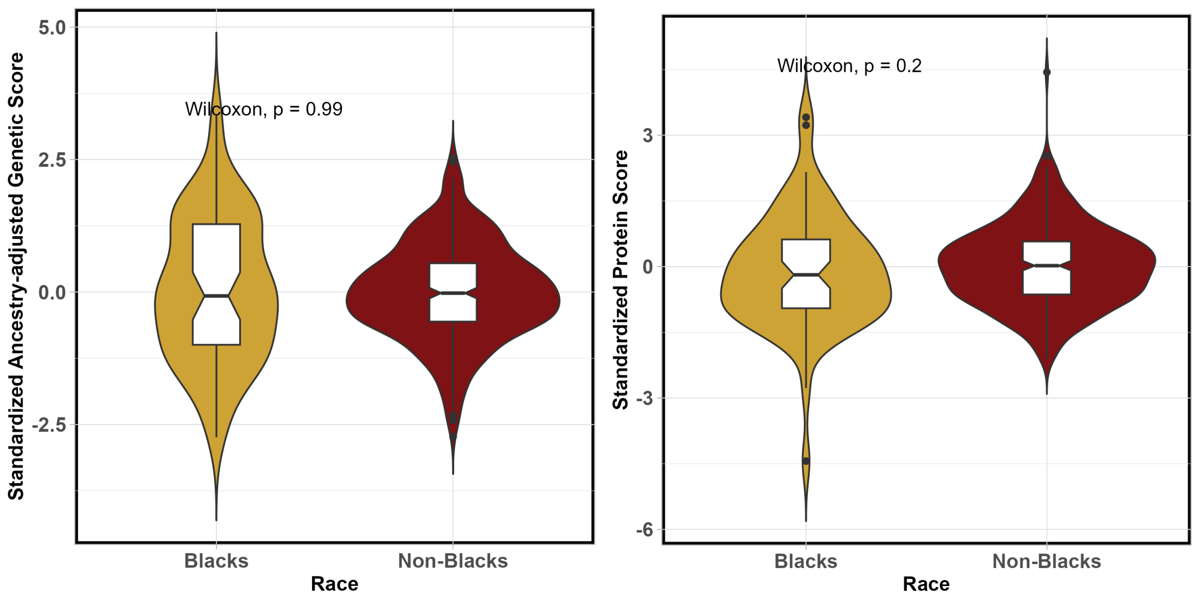

Wilcoxon, p = 0.99
5.0
2.5
0.0
-2.5
Standardized Ancestry-adjusted Genetic Score
Blacks
Non-Blacks
Race
Wilcoxon, p = 0.2
3
0
-3
-6
Standardized Protein Score
Blacks
Non-Blacks
Race

Figure S4 Correlation of standardized protein score and standardized proteins with age (A) and heatmap of p-values comparing the means of the score and signatures across variables (B). P-values are from ANOVA tests and * indicates statistically significant associations (P-Value < 0.05)

A

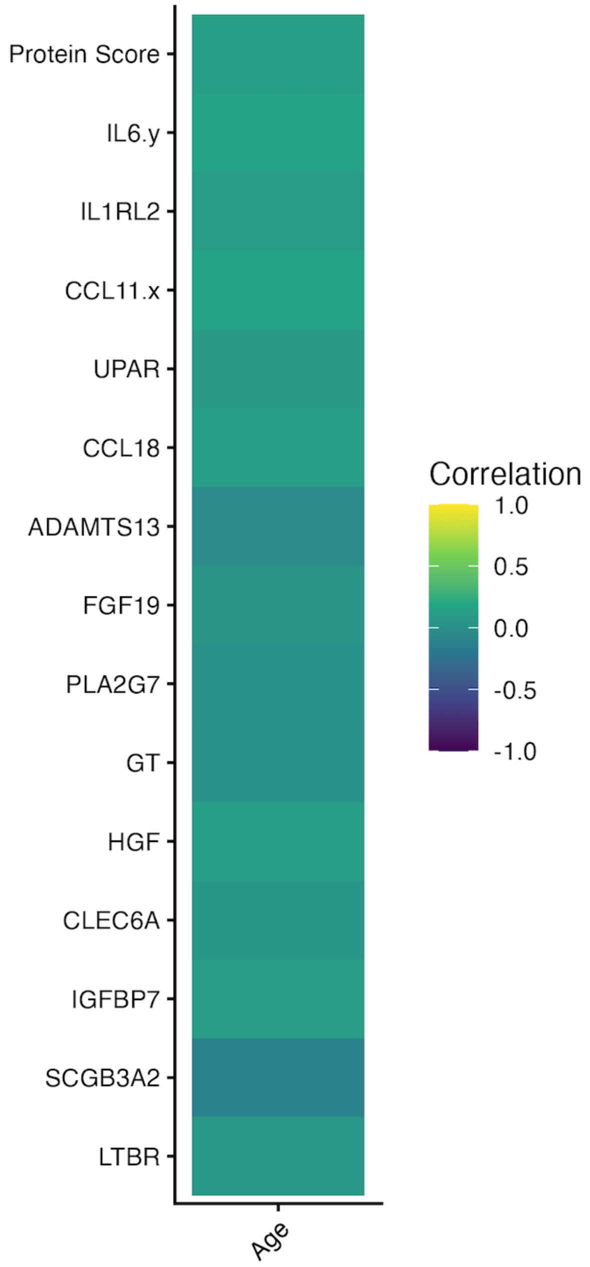

B

	Gender			Black Race			Diabetes Dx			Lipids Rx			BP Rx			CVD BL			ART BL		
	Male	Female	p-value	No	Yes	p-value	No	Yes	p-value	No	Yes	p-value	No	Yes	p-value	No	Yes	p-value	No	Yes	p-value
LTBR	4.65	4.58	0.33	4.64	4.62	0.66	4.63	4.74	0.21	4.63	4.64	0.85	4.61	4.69	0.21	4.64	4.72	0.45	4.72	4.60	0.02
SCGB3A2	3.64	3.40	0.19	3.59	3.70	0.44	3.61	3.50	0.62	3.64	3.47	0.25	3.63	3.51	0.42	3.59	3.97	0.16	3.49	3.66	0.18
IGFBP7	8.24	8.07	0.02	8.20	8.32	0.07	8.20	8.48	0.00	8.21	8.24	0.60	8.18	8.36	0.01	8.20	8.52	0.01	8.26	8.20	0.22
CLEC6A	2.78	2.84	0.58	2.81	2.65	0.12	2.79	2.73	0.72	2.77	2.70	0.49	2.74	2.82	0.44	2.77	3.03	0.16	3.19	2.60	0.00
HGF	8.78	8.62	0.05	8.77	8.71	0.41	8.74	9.04	0.00	8.75	8.89	0.04	8.75	8.86	0.12	8.75	9.10	0.01	8.67	8.81	0.02
GT	2.71	2.92	0.12	2.68	3.01	0.00	2.75	2.58	0.33	2.73	2.69	0.68	2.73	2.70	0.80	2.74	2.60	0.51	3.06	2.59	0.00
PLA2G7	2.18	1.85	0.00	2.21	1.81	0.00	2.15	1.99	0.09	2.13	2.20	0.29	2.16	2.08	0.21	2.14	2.10	0.67	1.93	2.24	0.00
FGF19	8.56	8.74	0.25	8.55	8.75	0.15	8.58	8.69	0.56	8.57	8.56	0.96	8.52	8.79	0.04	8.59	8.56	0.89	8.69	8.54	0.16
ADAMTS13	5.89	5.98	0.07	5.88	5.99	0.01	5.90	5.90	1.00	5.91	5.88	0.50	5.90	5.89	0.69	5.90	5.89	0.90	5.94	5.88	0.07
CCL18	7.44	7.39	0.73	7.43	7.45	0.87	7.41	7.76	0.08	7.35	7.77	0.00	7.44	7.40	0.77	7.43	7.55	0.63	7.27	7.51	0.03
UPAR	5.96	5.96	0.99	5.95	6.04	0.22	5.96	6.06	0.33	5.94	5.97	0.65	5.94	5.99	0.50	5.95	6.16	0.11	6.00	5.94	0.30
CCL11.x	8.50	8.35	0.05	8.47	8.53	0.38	8.46	8.79	0.00	8.48	8.48	0.98	8.46	8.57	0.09	8.47	8.80	0.01	8.63	8.41	0.00
IL1RL2	4.33	4.49	0.12	4.34	4.37	0.77	4.34	4.49	0.27	4.32	4.51	0.04	4.36	4.37	0.85	4.35	4.37	0.89	4.42	4.32	0.19
IL6.y	4.08	4.14	0.64	4.06	4.21	0.18	4.07	4.37	0.06	4.03	4.20	0.09	4.02	4.22	0.07	4.05	4.78	0.00	4.28	4.00	0.00
Protein Score	29.70	28.65	0.01	29.64	29.25	0.26	29.49	30.63	0.03	29.44	29.84	0.24	29.41	30.03	0.07	29.48	31.30	0.00	29.57	29.57	0.99

From Figures S3 and S4, the protein score and protein expressions are moderately correlated with age. The distribution of the protein score was not statistically different between individuals at or below 65 years and individuals above 65 and blacks and non-blacks. Being on ART or not, taking blood pressure treatment or not, and taking lipid treatment or not. However, the means differed statistically between males and females, between those with and without CVD at baseline, and those with diabetes vs those without. The finding for gender warrants further investigation given the limited sample size for females.

Figure S5 Correlation of standardized genetic score and standardized SNPs with age (A) and heatmap of p-values comparing the means of the score and genetic variants across variables (B).. Genetic score is not adjusted for ancestry. P-values are from ANOVA tests and * indicates statistically significant associations (P-Value < 0.05)

A

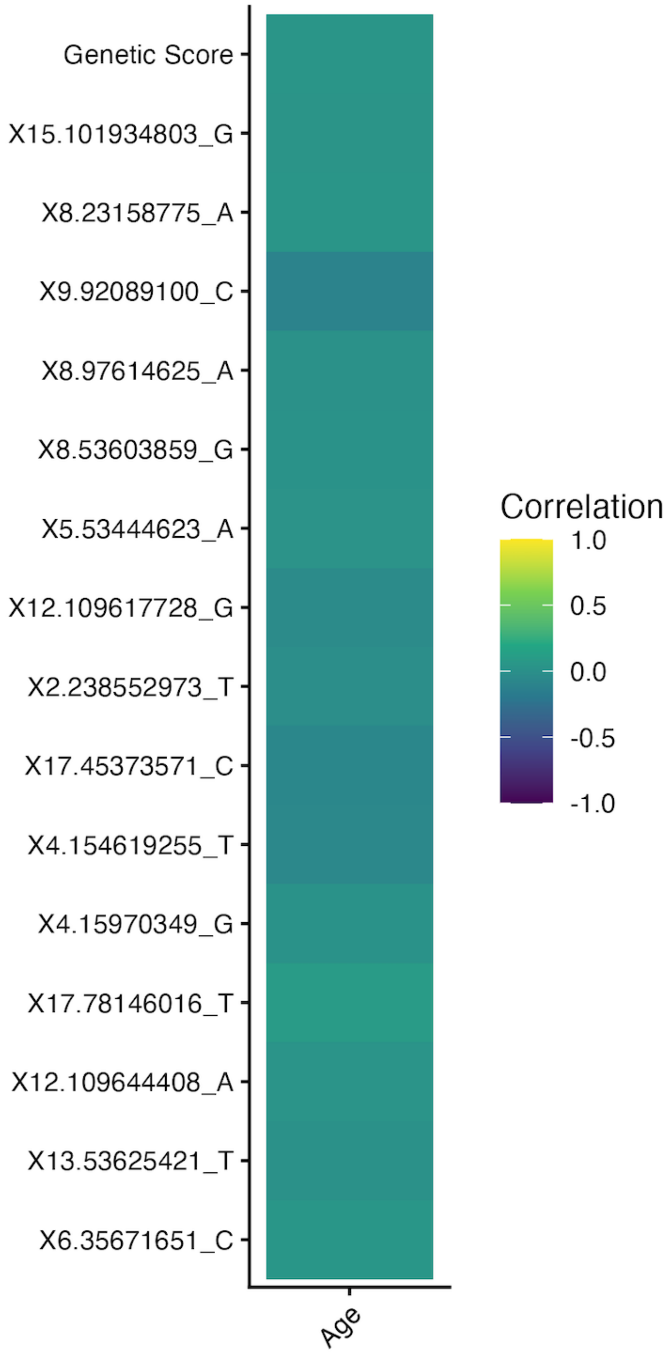

B

	Gender			Black Race			Diabetes Dx			Lipids Rx			BP Rx			CVD BL			ART BL		
	Male	Female	p-value	No	Yes	p-value	No	Yes	p-value	No	Yes	p-value	No	Yes	p-value	No	Yes	p-value	No	Yes	p-value
X6.35671651_C	0.39	0.37	0.86	0.37	0.46	0.23	0.38	0.48	0.36	0.39	0.37	0.81	0.39	0.38	0.98	0.38	0.44	0.65	0.43	0.36	0.32
X13.53625421_T	0.10	0.07	0.43	0.10	0.11	0.81	0.10	0.11	0.84	0.10	0.09	0.79	0.08	0.17	0.03	0.09	0.22	0.08	0.14	0.08	0.11
X12.109644408_A	0.16	0.11	0.41	0.15	0.17	0.64	0.15	0.19	0.61	0.15	0.14	0.91	0.15	0.14	0.83	0.16	0.00	0.07	0.15	0.15	0.89
X17.78146016_T	0.70	0.54	0.13	0.69	0.60	0.28	0.68	0.67	0.92	0.66	0.79	0.14	0.65	0.82	0.07	0.66	1.00	0.03	0.68	0.68	0.91
X4.15970349_G	0.45	0.26	0.03	0.48	0.15	0.00	0.43	0.33	0.38	0.45	0.36	0.21	0.45	0.35	0.21	0.43	0.44	0.89	0.40	0.44	0.57
X4.154619255_T	0.31	0.43	0.16	0.20	0.89	0.00	0.32	0.41	0.43	0.31	0.30	0.85	0.29	0.38	0.24	0.33	0.28	0.72	0.47	0.26	0.00
X17.45373571_C	0.32	0.54	0.01	0.27	0.74	0.00	0.35	0.30	0.62	0.34	0.33	0.83	0.33	0.38	0.49	0.35	0.39	0.75	0.46	0.30	0.01
X2.238552973_T	0.17	0.20	0.66	0.12	0.40	0.00	0.17	0.15	0.75	0.16	0.19	0.59	0.15	0.25	0.05	0.16	0.39	0.02	0.19	0.16	0.58
X12.109617728_G	0.10	0.35	0.00	0.06	0.49	0.00	0.11	0.44	0.00	0.12	0.14	0.69	0.11	0.23	0.01	0.12	0.33	0.02	0.21	0.10	0.01
X5.53444623_A	0.13	0.15	0.68	0.10	0.26	0.00	0.13	0.19	0.42	0.13	0.11	0.72	0.12	0.17	0.29	0.13	0.17	0.67	0.16	0.12	0.26
X8.53603859_G	0.21	0.13	0.24	0.23	0.03	0.00	0.20	0.19	0.87	0.21	0.16	0.32	0.20	0.22	0.79	0.19	0.33	0.16	0.21	0.19	0.63
X8.97614625_A	0.12	0.09	0.56	0.11	0.12	0.77	0.12	0.07	0.51	0.12	0.10	0.68	0.12	0.09	0.55	0.11	0.17	0.46	0.09	0.12	0.26
X9.92089100_C	0.50	0.54	0.62	0.51	0.48	0.73	0.51	0.37	0.26	0.50	0.54	0.58	0.53	0.40	0.13	0.49	0.67	0.25	0.46	0.52	0.40
X8.23158775_A	0.63	0.50	0.19	0.66	0.38	0.00	0.63	0.41	0.08	0.62	0.63	0.89	0.60	0.69	0.31	0.61	0.72	0.46	0.51	0.66	0.04
X15.101934803_G	0.22	0.30	0.28	0.18	0.51	0.00	0.22	0.41	0.05	0.22	0.26	0.60	0.21	0.31	0.15	0.23	0.39	0.15	0.31	0.20	0.04
SNP Score	0.53	0.59	0.79	0.40	1.18	0.00	0.51	0.85	0.30	0.49	0.60	0.63	0.39	1.08	0.00	0.48	1.56	0.01	0.66	0.48	0.31

From Figure S5, the unadjusted genetic score is moderately correlated with age (A). From (B), the mean of the genetic score was not statistically different between individuals on ART vs those not on ART, diabetes or not, taking lipid treatment or not and between males and females. However, the means differed statistically between blacks and non-blacks, those with and without CVD at baseline, and those on blood pressure treatment at baseline.

References

1. Feinstein, M. J. HIV and Cardiovascular Disease: From Insights to Interventions. *Top. Antivir. Med.* **29**, 407–411 (2021).
2. HIV and Heart Disease | NIH. https://hivinfo.nih.gov/understanding-hiv/fact-sheets/hiv-and-heart-disease.
3. Shah, A. S. V. *et al.* Global Burden of Atherosclerotic Cardiovascular Disease in People Living With HIV. *Circulation* **138**, 1100–1112 (2018).
4. Feinstein, M. J. *et al.* Characteristics, Prevention, and Management of Cardiovascular Disease in People Living With HIV: A Scientific Statement From the American Heart Association. *Circulation* **140**, e98–e124 (2019).
5. Freiberg, M. S. *et al.* HIV Infection and the Risk of Acute Myocardial Infarction. *JAMA Intern. Med.* **173**, 614–622 (2013).
6. Drozd, D. R. *et al.* Increased Risk of Myocardial Infarction in HIV-Infected Individuals in North America Compared to the General Population. *J. Acquir. Immune Defic. Syndr. 1999* **75**, 568–576 (2017).
7. Feinstein, M. J. *et al.* Adjudicated Heart Failure in HIV-Infected and Uninfected Men and Women. *J. Am. Heart Assoc.* **7**, e009985 (2018).
8. Achhra, A. C. *et al.* Assessing Cardiovascular Risk in People Living with HIV: Current Tools and Limitations. *Curr. HIV/AIDS Rep.* **18**, 271–279 (2021).
9. Rao, A. S. & Knowles, J. W. Polygenic risk scores in coronary artery disease. *Curr. Opin. Cardiol.* **34**, 435 (2019).
10. O'Sullivan, J. W. *et al.* Polygenic Risk Scores for Cardiovascular Disease: A Scientific Statement From the American Heart Association. *Circulation* **146**, e93–e118 (2022).

11. Safo, S. E., Min, E. J. & Haine, L. Sparse linear discriminant analysis for multiview structured data. *Biometrics* **78**, 612–623 (2022).

12. MacArthur, R. D. *et al.* A comparison of three highly active antiretroviral treatment strategies consisting of non-nucleoside reverse transcriptase inhibitors, protease inhibitors, or both in the presence of nucleoside reverse transcriptase inhibitors as initial therapy (CPCRA 058 FIRST Study): a long-term randomised trial. *Lancet Lond. Engl.* **368**, 2125–2135 (2006).

13. Interleukin-2 Therapy in Patients with HIV Infection. *N. Engl. J. Med.* **361**, 1548–1559 (2009).

14. Strategies for Management of Antiretroviral Therapy (SMART) Study Group *et al.* CD4+ count-guided interruption of antiretroviral treatment. *N. Engl. J. Med.* **355**, 2283–2296 (2006).

15. Initiation of Antiretroviral Therapy in Early Asymptomatic HIV Infection. *N. Engl. J. Med.* **373**, 795–807 (2015).

16. Olink Target 96 Cardiovascular panels - Olink. https://olink.com/products-services/target/cardiometabolic-panel/.

17. Krämer, A., Green, J., Pollard, J., Jr & Tugendreich, S. Causal analysis approaches in Ingenuity Pathway Analysis. *Bioinformatics* **30**, 523–530 (2014).

18. STRING: functional protein association networks. https://string-db.org/.

19. Kerr, K. F. *et al.* Net Reclassification Indices for Evaluating Risk-Prediction Instruments: A Critical Review. *Epidemiol. Camb. Mass* **25**, 114–121 (2014).

20. Pencina, M. J., D'Agostino, R. B. & Steyerberg, E. W. Extensions of net reclassification improvement calculations to measure usefulness of new biomarkers. *Stat. Med.* **30**, 11–21 (2011).

21. Pencina, M. J., D'Agostino, R. B., D'Agostino, R. B. & Vasan, R. S. Evaluating the added predictive ability of a new marker: from area under the ROC curve to reclassification and beyond. *Stat. Med.* **27**, 157–172; discussion 207-212 (2008).

22. PhenoScanner. http://www.phenoscanner.medschl.cam.ac.uk/.

23. Boehm, F. J. & Zhou, X. Statistical methods for Mendelian randomization in genome-wide association studies: A review. *Comput. Struct. Biotechnol. J.* **20**, 2338–2351 (2022).

24. Safo, S. E. *et al.* Derivation of a Protein Risk Score for Cardiovascular Disease Among a Multiracial and Multiethnic HIV+ Cohort. *J. Am. Heart Assoc.* **12**, e027273 (2023).

25. Duprez, D. A. *et al.* Inflammation, Coagulation and Cardiovascular Disease in HIV-Infected Individuals. *PLoS ONE* **7**, e44454 (2012).

26. Nordell, A. D. *et al.* Severity of Cardiovascular Disease Outcomes Among Patients With HIV Is Related to Markers of Inflammation and Coagulation. *J. Am. Heart Assoc. Cardiovasc. Cerebrovasc. Dis.* **3**, e000844 (2014).

27. HSU, D. C. *et al.* Plasma IL-6 levels are independently associated with atherosclerosis and mortality in HIV-infected individuals on suppressive ART. *AIDS Lond. Engl.* **30**, 2065–2074 (2016).

28. Hurt-Camejo, E., Camejo, G., Peilot, H., Oörni, K. & Kovanen, P. Phospholipase A(2) in vascular disease. *Circ. Res.* **89**, 298–304 (2001).

29. Bell, E. J. *et al.* Hepatocyte growth factor is associated with progression of atherosclerosis: the Multi-Ethnic Study of Atherosclerosis (MESA). *Atherosclerosis* **272**, 162–167 (2018).

30. Bielinski, S. J. *et al.* Hepatocyte growth factor demonstrates racial heterogeneity as a biomarker for coronary heart disease. *Heart Br. Card. Soc.* **103**, 1185–1193 (2017).

31. Caslake, M. J. & Packard, C. J. Lipoprotein-associated phospholipase A2 (platelet-activating factor acetylhydrolase) and cardiovascular disease. *Curr. Opin. Lipidol.* **14**, 347–352 (2003).

32. Reilly, C. S. *et al.* Investigation of Causal Effects of Protein Biomarkers on Cardiovascular Disease in Persons With HIV. *J. Infect. Dis.* **227**, 951–960 (2023).

33. Fourman, L. T. *et al.* Proteomic Analysis of Hepatic Fibrosis in Human Immunodeficiency Virus-Associated Nonalcoholic Fatty Liver Disease Demonstrates Up-regulation of Immune Response and Tissue Repair Pathways. *J. Infect. Dis.* **227**, 565–576 (2023).

34. Thumser, A. E., Moore, J. B. & Plant, N. J. Fatty acid binding proteins: tissue-specific functions in health and disease. *Curr. Opin. Clin. Nutr. Metab. Care* **17**, 124–129 (2014).

35. Inoue, K. *et al.* Plasma UGRP1 levels associate with promoter G-112A polymorphism and the severity of asthma. *Allergol. Int. Off. J. Jpn. Soc. Allergol.* **57**, 57–64 (2008).

36. Orekhov, A. N. *et al.* Tumor Necrosis Factor-α and C-C Motif Chemokine Ligand 18 Associate with Atherosclerotic Lipid Accumulation In situ and In vitro. *Curr. Pharm. Des.* **24**, 2883–2889 (2018).

37. Subramanian, N. *et al.* Adipose tissue specific CCL18 associates with cardiometabolic diseases in non-obese individuals implicating CD4+ T cells. *Cardiovasc. Diabetol.* **22**, 84 (2023).

38. Emanuele, E. *et al.* Association of plasma eotaxin levels with the presence and extent of angiographic coronary artery disease. *Atherosclerosis* **186**, 140–145 (2006).

39. Gutiérrez-Rivas, M. *et al.* High Plasma Levels of sTNF-R1 and CCL11 Are Related to CD4+ T-Cells Fall in Human Immunodeficiency Virus Elite Controllers With a Sustained Virologic Control. *Front. Immunol.* **9**, 1399 (2018).

40. Ganz, P. *et al.* Development and Validation of a Protein-Based Risk Score for Cardiovascular Outcomes Among Patients With Stable Coronary Heart Disease. *JAMA* **315**, 2532–2541 (2016).

41. Riveros-Mckay, F. *et al.* Integrated Polygenic Tool Substantially Enhances Coronary Artery Disease Prediction. *Circ. Genomic Precis. Med.* **14**, e003304 (2021).

42. Tang, X.-D. *et al.* Pathogenesis and Treatment of Cytokine Storm Induced by Infectious Diseases. *Int. J. Mol. Sci.* **22**, 13009 (2021).

43. Ferrari, R. The role of TNF in cardiovascular disease. *Pharmacol. Res.* **40**, 97–105 (1999).

44. Yuan, S. *et al.* Effects of tumour necrosis factor on cardiovascular disease and cancer: A two-sample Mendelian randomization study. *eBioMedicine* **59**, (2020).

45. Gallucci, G., Tartarone, A., Lerose, R., Lalinga, A. V. & Capobianco, A. M. Cardiovascular risk of smoking and benefits of smoking cessation. *J. Thorac. Dis.* **12**, 3866–3876 (2020).

46. CDCTobaccoFree. People Living With HIV - Tips From Former Smokers. *Centers for Disease Control and Prevention* https://www.cdc.gov/tobacco/campaign/tips/groups/hiv.html (2023).

47. Mdodo, R. *et al.* Cigarette smoking prevalence among adults with HIV compared with the general adult population in the United States: cross-sectional surveys. *Ann. Intern. Med.* **162**, 335–344 (2015).

48. PubChem. FGF19 - fibroblast growth factor 19 (human). https://pubchem.ncbi.nlm.nih.gov/gene/FGF19/human.

49. Degirolamo, C., Sabbà, C. & Moschetta, A. Therapeutic potential of the endocrine fibroblast growth factors FGF19, FGF21 and FGF23. *Nat. Rev. Drug Discov.* **15**, 51–69 (2016).

50. Fisher, E. *et al.* Evidence for the Thr79Met polymorphism of the ileal fatty acid binding protein (FABP6) to be associated with type 2 diabetes in obese individuals. *Mol. Genet. Metab.* **98**, 400–405 (2009).

51. Maino, A. *et al.* Plasma ADAMTS-13 levels and the risk of myocardial infarction: an individual patient data meta-analysis. *J. Thromb. Haemost. JTH* **13**, 1396–1404 (2015).

52. Graham, S. M. *et al.* Von Willebrand Factor Adhesive Activity and ADAMTS13 Protease Activity in HIV-1-Infected Men. *Int. J. Med. Sci.* **16**, 276–284 (2019).

53. Kaneko, N., Kurata, M., Yamamoto, T., Morikawa, S. & Masumoto, J. The role of interleukin-1 in general pathology. *Inflamm. Regen.* **39**, 12 (2019).

54. Abbate, A. *et al.* Interleukin-1 and the Inflammasome as Therapeutic Targets in Cardiovascular Disease. *Circ. Res.* **126**, 1260–1280 (2020).

55. Kolossváry, M. *et al.* Proteomic Signature of Subclinical Coronary Artery Disease in People With HIV: Analysis of the REPRIEVE Mechanistic Substudy. *J. Infect. Dis.* **226**, 1809–1822 (2022).

56. Baker, S. K. & Strickland, S. A critical role for plasminogen in inflammation. *J. Exp. Med.* **217**, e20191865 (2020).

57. Mahmood, N., Mihalcioiu, C. & Rabbani, S. A. Multifaceted Role of the Urokinase-Type Plasminogen Activator (uPA) and Its Receptor (uPAR): Diagnostic, Prognostic, and Therapeutic Applications. *Front. Oncol.* **8**, 24 (2018).

58. Olson, N. C. *et al.* Soluble Urokinase Plasminogen Activator Receptor: Genetic Variation and Cardiovascular Disease Risk in Black Adults. *Circ. Genomic Precis. Med.* **14**, e003421 (2021).

59. Kirkegaard-Klitbo, D. M. *et al.* Soluble Urokinase Plasminogen Activator Receptor Is a Predictor of Incident Non-AIDS Comorbidity and All-Cause Mortality in Human Immunodeficiency Virus Type 1 Infection. *J. Infect. Dis.* **216**, 819–823 (2017).

60. Zhou, Z. *et al.* Uterus globulin associated protein 1 (UGRP1) is a potential marker of progression of Graves' disease into hypothyroidism. *Mol. Cell. Endocrinol.* **494**, 110492 (2019).

61. Jin, L., Shen, F., Weinfeld, M. & Sergi, C. Insulin Growth Factor Binding Protein 7 (IGFBP7)-Related Cancer and IGFBP3 and IGFBP7 Crosstalk. *Front. Oncol.* **10**, (2020).

62. Lisowska, A. *et al.* IGFBP7 Concentration May Reflect Subclinical Myocardial Damage and Kidney Function in Patients with Stable Ischemic Heart Disease. *Biomolecules* **12**, 274 (2022).

63. Higashi, Y., Gautam, S., Delafontaine, P. & Sukhanov, S. IGF-1 and cardiovascular disease. *Growth Horm. IGF Res. Off. J. Growth Horm. Res. Soc. Int. IGF Res. Soc.* **45**, 6–16 (2019).
