# Supplementary material for: Proteomic and genetic predictors and risk scores of cardiovascular diseases in persons living with HIV
Source: Front Cardiovasc Med. 2026 Jan 28;12:1726272. doi: 10.3389/fcvm.2025.1726272 (PMC12892344; doi:10.3389/fcvm.2025.1726272)
Supplement: Supplementary file 2 [file Datasheet2.pdf]

Supplementary Appendix

This appendix has been provided by the authors to give readers additional information about their work.

Supplement to: The INSIGHT–ESPRIT Study Group and SILCAAT Scientific Committee. Interleukin-2 therapy in patients with HIV infection. N Engl J Med 2009;361:1548-59.

Design and Methods for SILCAAT and ESPRIT

CD4+ Goals

SILCAAT

After the induction phase, additional cycles of IL-2 therapy were to be given in order to maintain the CD4+ cell count greater than a threshold of 175 cells above baseline for those with a baseline CD4+ count of 200 to 299 cells/mm^3 and 125 cells above baseline for those with a CD4+ count of 50-199 cells/mm^3 at study entry.

ESPRIT

After the induction phase, additional cycles of IL-2 therapy were to be given in order to maintain the CD4+ cell count greater than twice baseline or ≥1000 cells/mm^3.

Study Design

SILCAAT

Patients were randomized to IL-2 or control (1:1) within strata defined by clinical site and CD4+ cell count (50-199 and 200-299 cells/mm^3) (Web Figure 1a).

The original design of SILCAAT specified a target of 210 primary events and assumed a CD4+ cell count difference between treatment groups of 75 cells/mm^3. At the time of the change in sponsorship and amendment in February 2003 the target number of primary events was increased to 300 because some deaths occurring as primary events would be unrelated to HIV and IL-2 and this was expected to reduce the treatment difference. With 300 primary events, power was 80% to detect a 28% reduction in the rate of opportunistic disease or death between the two treatment groups (hazard ratio of 0.72) with a two-sided alpha level of 0.05.

ESPRIT

Patients were randomized to IL-2 or control (1:1) within strata defined by clinical site (Web Figure 1b).

For ESPRIT, we calculated that 320 primary events would be required to have statistical power of 80% to detect a 27% reduction in the rate of opportunistic disease or death between the two treatment groups (hazard ratio of 0.73) with a two-sided alpha level of 0.05. The hypothesized treatment effect took into account the projected CD4+ difference between the IL-2 and control group (140 cells/mm^3) and the likely proportion of deaths unrelated to HIV and to IL-2 (10% of primary events). During the study, it became apparent that non-AIDS deaths made up a larger proportion of primary endpoints than estimated and the overall event rate was lower than anticipated. Consideration was given to re-powering the study, but in light of data from other sources indicating that the risk of non-AIDS deaths was inversely related to latest CD4+ cell count levels, the target number of primary events was not modified.

Inclusion and Exclusion Criteria

SILCAAT

Patients in SILCAAT were ≥18 years with confirmed HIV-1 infection, CD4+ cell count ≥ 50 cells/mm^3 and < 300 cells/mm^3, HIV RNA level < 10,000 copies/mL, and on stable ART (≥ 2 drugs for ≥ 4 months prior to randomization, with no change in the type of ART received during this 4-month period of time). The main exclusion criteria were a recent history of an AIDS-defining illness, evidence of active infection, any significant medical condition, current pregnancy, breastfeeding, and use of corticosteroids or immunosuppressants.

After the change in study management and amendment of the protocol in 2003 some patients chose not to re-consent for the study and some sites were no longer able to participate due to

reduced compensation. Some of the patients from sites that discontinued participation were transferred to other sites for the remainder of the trial, but for many patients this was not possible. The following algorithm was then used to define the primary analysis cohort: all randomized patients at sites for which at least two-thirds (67%) of their patients consented to the amended protocol or had died were included in the primary analysis. This definition of the primary analysis cohort represented a compromise between controlling bias due to possible selective and differential lost to follow-up by treatment group and maintaining power by including a large enough sample size to address the study objectives (Web Figure 1a).

ESPRIT

Patients in ESPRIT were ≥18 years with confirmed HIV-1 infection, CD4+ cell count ≥ 300 cells/mm^3 and no evidence of active clinical disease for at least one year prior to randomization. The main exclusion criteria for ESPRIT were current or prior autoimmune disease, including inflammatory bowel disease, current pregnancy, breastfeeding or use of corticosteroids and/or immunosuppressants.

ESPRIT was preceded by four randomized phase II trials at sites in Argentina, Thailand, and the United States. These trials (vanguard studies) had CD4+ change as the primary endpoint rather than clinical disease progression. Patients in these vanguard studies were invited to enroll in ESPRIT and new consent was obtained. Sites where at least 90% of the vanguard patients consented to ESPRIT were included in the primary analysis (Web Figure 1b). In three of the vanguard studies, entry CD4+ cell count was ≥ 350 cells/mm^3. In each of these vanguard studies, some patients were assigned a lower dose of IL-2 (1.5 or 4.5 MIU) for the induction phase. These patients were encouraged but not required to escalate to the 7.5 MIU dose.

Endpoint Definitions

For both ESPRIT and SILCAAT the following new or recurrent opportunistic diseases were included in the primary endpoint were: invasive aspergillosis; bartonellosis; esophageal candidiasis; candidiasis of bronchi, trachea, or lungs; invasive cervical cancer; Chagas disease of the central nervous system; cytomegalovirus virus (CMV) disease; CMV retinitis; disseminated or extrapulmonary coccidioidomycosis; chronic intestinal cryptosporidiosis; extrapulmonary cryptococcosis; HIV-related encephalopathy; *Herpes simplex,* chronic ulcers, bronchitis, pneumonitis, or esophagitis; *Herpes zoster,* multi-dermatomal; disseminated or extrapulmonary histoplasmosis; chronic intestinal isosporiasis; Kaposi's sarcoma; visceral leishmaniasis; Burkitt's lymphoma; Hodgkin's lymphoma; non-Hodgkin's lymphoma; primary lymphoma of the brain; tuberculosis; microsporidiosis (1 month's duration); disseminated or extrapulmonary *Mycobacterium avium* complex or *M. kansasii*; other disseminated or extrapulmonary non-tuberculous mycobacterial species or unidentified species; nocardiosis; disseminated *Penicillium marneffei*; extrapulmonary *Pneumocystis jirovecii*; *Pneumocystis jirovecii* pneumonia; recurrent bacterial pneumonia (2 episodes within 1 year of each other following randomization); progressive multifocal leukoencephalopathy; *Rhodococcus equi* disease; recurrent *Salmonella* septicemia (2 episodes within 1 year of each other following randomization); toxoplasmosis of brain; wasting syndrome due to HIV.

Using pre-established criteria, an endpoint review committee that was unaware of the treatment assignment reviewed the source documentation of events reported as opportunistic diseases and all deaths. Opportunistic diseases classified as confirmed or probable were considered primary endpoints, as were all deaths, irrespective of cause. For patients who died, the endpoint review committee also classified the underlying cause of death using the Coding of Death in HIV (CoDe) system (http://www.cphiv.dk) .

Interim Monitoring

For both ESPRIT and SILCAAT, O'Brien-Fleming boundaries and the Lan-DeMets spending function were used as monitoring guidelines for the primary endpoint comparison.

For ESPRIT, futility guidelines were also provided to the DSMB. Since the beneficial effects of IL-2 might not be realized until later in follow-up, a planned futility analysis was to be carried out when 200 of 320 planned primary events had accrued. As a guideline, the (DSMB) was asked to assess whether at least a 10% lower rate of the primary endpoint in the IL-2 group compared to the control group was likely.

Web Figure Legends

Web Figure Ia: SILCAAT Study Design and CONSORT Flow Diagram. Endpoint status was considered known if the patient had been seen in the previous year.

Web Figure Ib: ESPRIT Study Design and CONSORT Flow Diagram. Endpoint status was considered known if the patient had been seen in the previous year.

Web Figure IIa: SILCAAT Study: Percent with HIV RNA ≤ 500 copies/mL by Follow-up Visit and Treatment Group.

Web Figure IIb: ESPRIT Study: Percent with HIV RNA ≤ 500 copies/mL by Follow-up Visit and Treatment Group.

Web Figure III: Cumulative Probability (x100) of Fatal or Non-Fatal OD by Treatment Group for SILCAAT and ESPRIT.

Web Figure IV: Cumulative Probability (x100) of Grade 4 Events by Treatment Group for SILCAAT and ESPRIT.

Web Figure Ia. SILCAAT study design and consort flow diagram

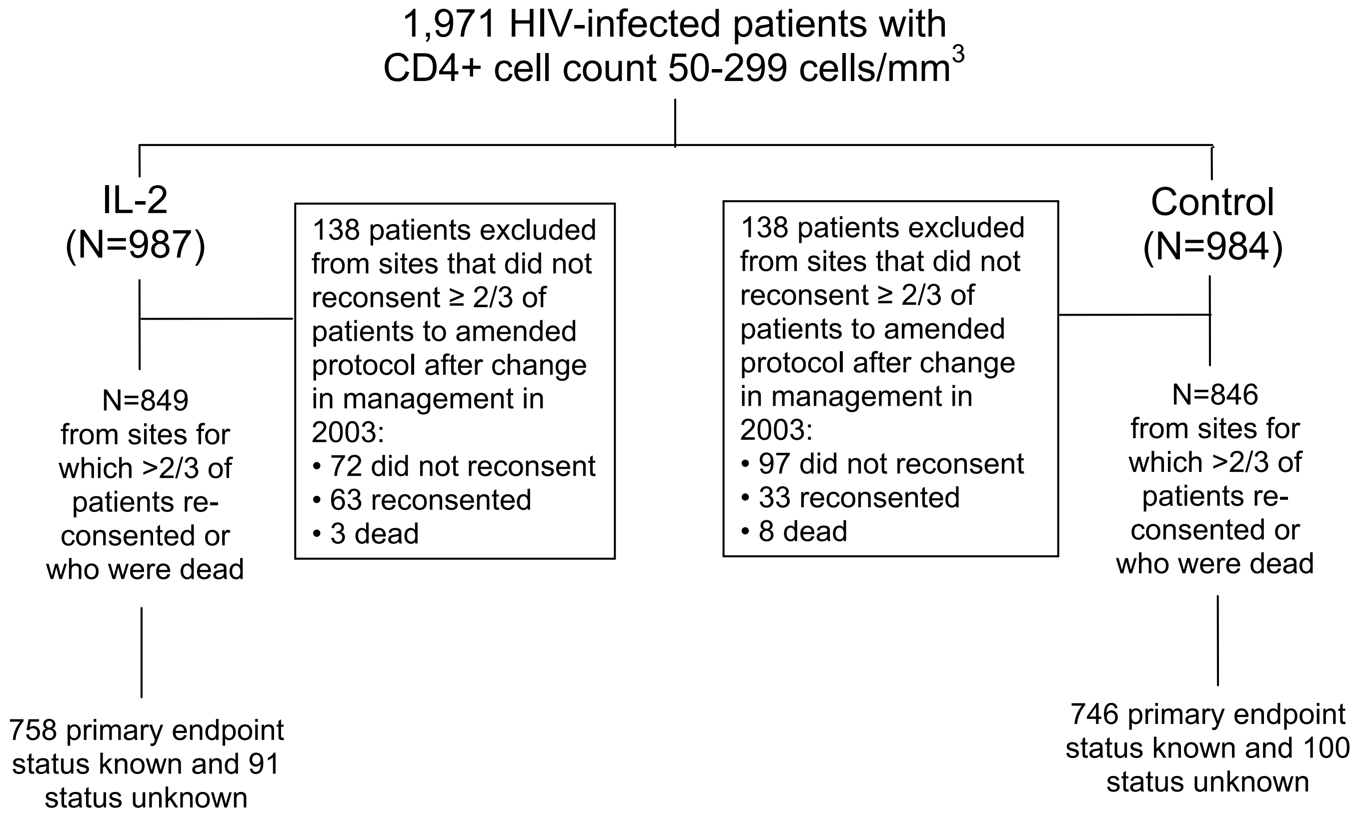

Web Figure Ib. ESPRIT study design and CONSORT flow diagram

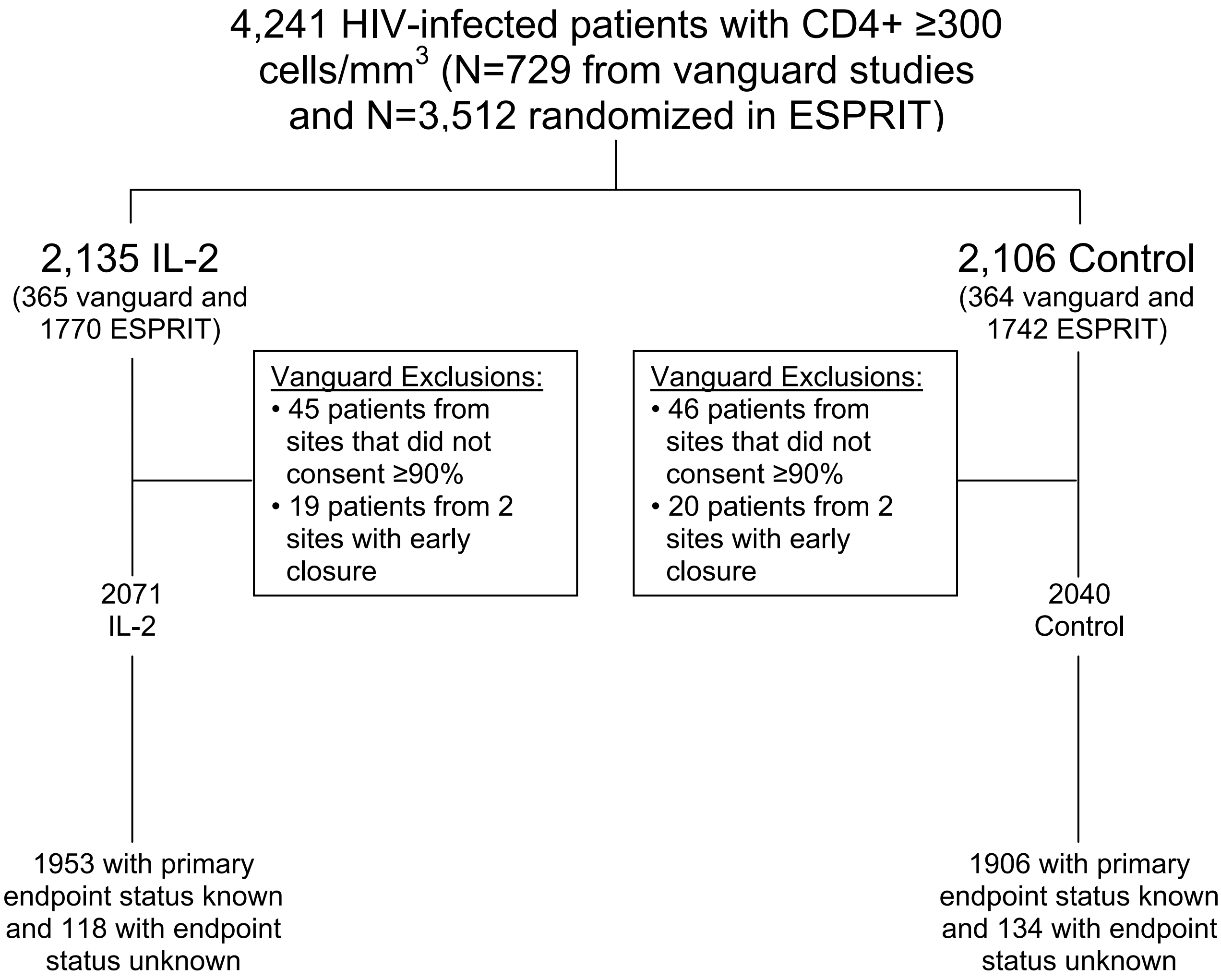

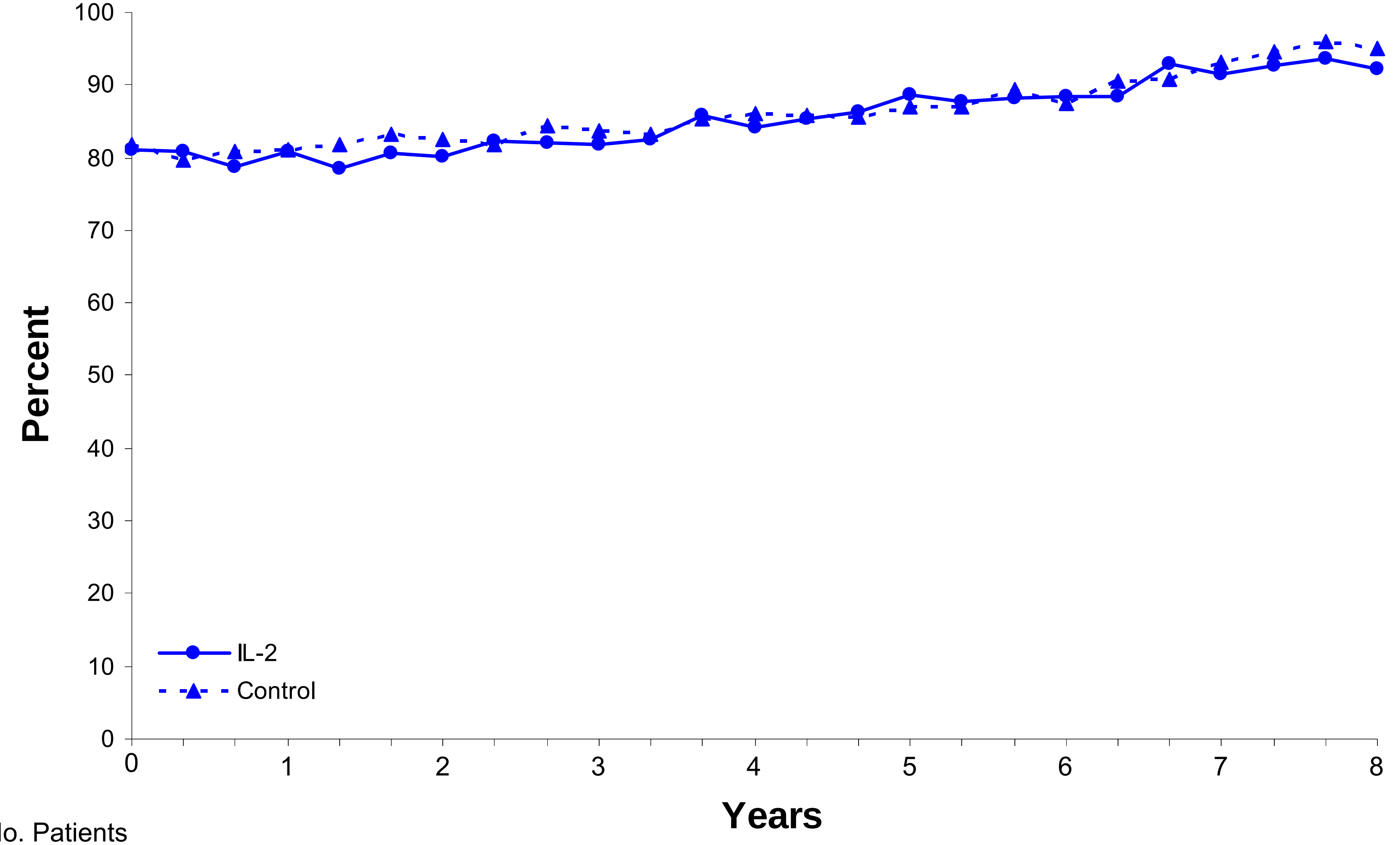

No. Patients

IL-2:	849	744	669	644	646	621	597	440	244
Control:	845	767	688	661	641	633	597	444	223

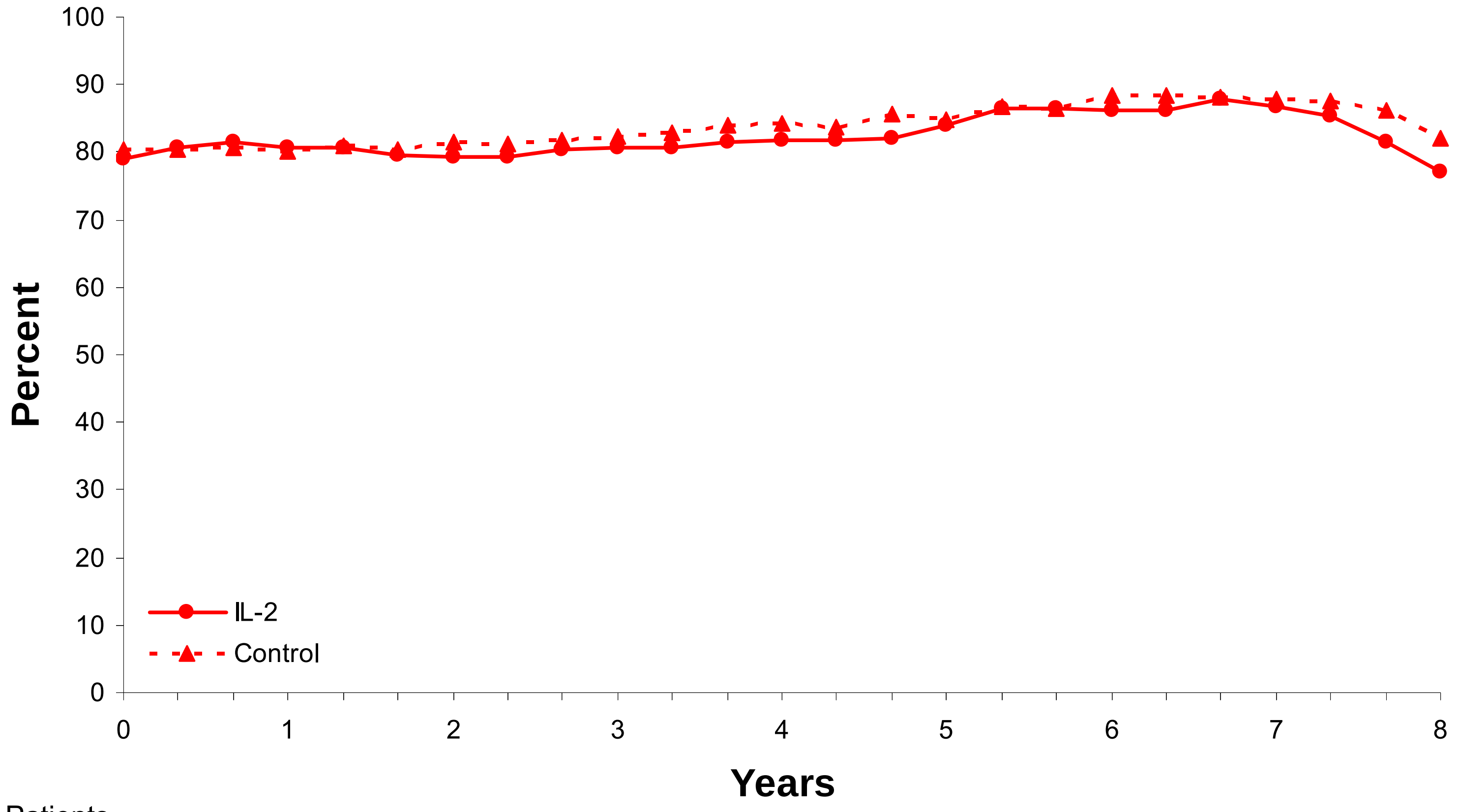

No. Patients

IL-2:	2065	1944	1864	1828	1774	1735	1419	894	301
Control:	2036	1921	1857	1791	1725	1643	1351	828	252

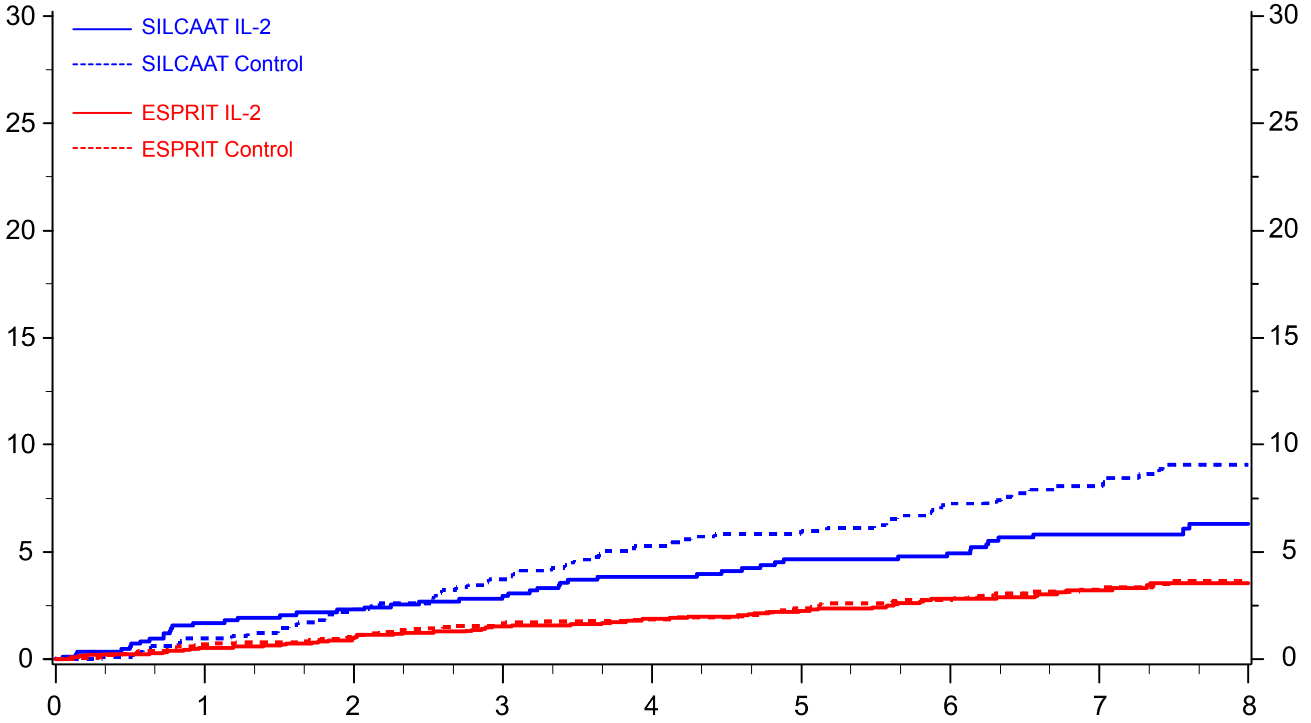

SILCAAT IL-2:	*849*	*807*	*779*	*757*	*726*	*698*	*683*	*497*	*257*
SILCAAT Control:	*846*	*810*	*779*	*746*	*706*	*679*	*651*	*494*	*247*
ESPRIT IL-2:	*2071*	*2030*	*1998*	*1948*	*1910*	*1874*	*1553*	*974*	*322*
ESPRIT Control:	*2040*	*2003*	*1963*	*1919*	*1884*	*1825*	*1486*	*909*	*271*

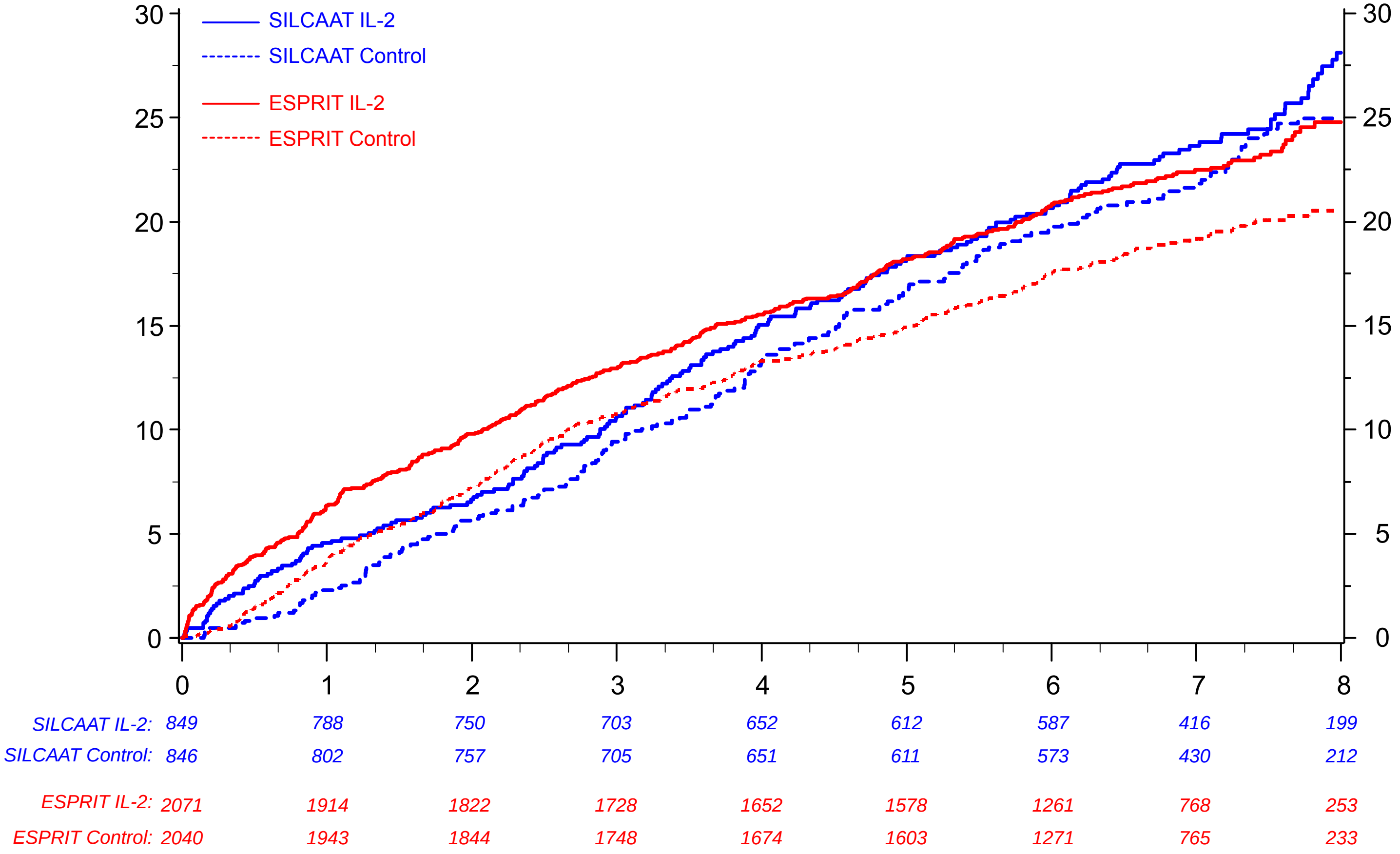
SILCAAT IL-2
SILCAAT Control
ESPRIT IL-2
ESPRIT Control
0
5
10
15
20
25
30
0
1
2
3
4
5
6
7
8
SILCAAT IL-2: 849 788 750 703 652 612 587 416 199
SILCAAT Control: 846 802 757 705 651 611 573 430 212
ESPRIT IL-2: 2071 1914 1822 1728 1652 1578 1261 768 253
ESPRIT Control: 2040 1943 1844 1748 1674 1603 1271 765 233

Web Table Ia. Baseline Characteristics of Participants by Treatment Group - SILCAAT

Characteristic	IL-2 Group (N = 849)	Control Group (N = 846)
Age (yr)		
Median	40	40
Interquartile range	36, 47	36, 47
Female sex (%)	16.1	16.9
Race (%)		
Black	7.8	9.1
White	80.0	79.6
Other	12.2	11.3
CD4+ count (cells/mm^3)		
Median	202	202
Interquartile range	154, 254	148, 255
CD4+ nadir (cells/mm^3)		
Median	59	60
Interquartile range	24, 104	28, 108
HIV RNA $\leq$ 500 (copies/mL (%)	81.0	81.8
AIDS diagnosis (%)	33.7	31.3
Body mass index (kg/m^2)		
Median	24.0	23.8
Interquartile range	21.9, 26.2	21.7, 25.9
Antiretroviral history (%)		
PI experience	85.7	85.2
NNRTI experience	58.8	56.6
NRTI, PI, and NNRTI experience	45.3	43.3
Time since first prescribed antiretroviral drugs (yr)		
Median	3.9	4.0
Interquartile range	1.7, 6.9	1.9, 7.4
Current antiretroviral regimen (%)		
Includes PI	67.7	63.3
Includes NNRTI	45.5	46.2
Includes NRTI, PI, and NNRTI	15.0	12.9

Web Table Ib. Baseline Characteristics of Participants by Treatment Group - ESPRIT

Characteristic	IL-2 Group (N = 2071)	Control Group (N = 2040)
Age (yr)		
Median	40	40
Interquartile range	35, 46	34, 46
Female sex (%)	18.6	18.7
Race (%)		
Black	9.5	8.8
White	75.1	75.5
Other	15.5	15.6
CD4+ count (cells/mm^3)		
Median	464	450
Interquartile range	373, 590	370, 576
CD4+ nadir (cells/mm^3)		
Median	200	194
Interquartile range	93, 308	90, 304
HIV RNA $\leq$ 500 (copies/mL (%)	79.0	80.4
AIDS diagnosis (%)	25.2	26.5
Body mass index (kg/m^2)		
Median	23.8	23.7
Interquartile range	21.9, 26.0	21.9, 25.9
Antiretroviral history (%)		
PI experience	72.8	72.1
NNRTI experience	56.5	59.4
NRTI, PI, and NNRTI experience	37.4	39.6
Time since first prescribed antiretroviral drugs (yr)		
Median	4.1	4.3
Interquartile range	2.2, 6.3	2.1, 6.5
Current antiretroviral regimen (%)		
Includes PI	50.5	47.5
Includes NNRTI	44.7	47.9
Includes NRTI, PI, and NNRTI	7.7	8.1

Web Table IIa: Rate of Specific Opportunistic Diseases by Treatment Group - SILCAAT

	IL-2		Control	
**Event**	**N**	**Rate**	**N**	**Rate**
Aspergillosis	0	0.00	2	0.03
Bacterial pneumonia, recurrent	3	0.05	4	0.07
Bartonellosis	0	0.00	0	0.00
Candidiasis of the bronchi/trachea/lungs	0	0.00	0	0.00
Candidiasis esophageal	10	0.17	19	0.33
Cervical carcinoma*	0	0.00	0	0.00
Chagas disease of the CNS	0	0.00	0	0.00
CMV disease	0	0.00	2	0.03
Coccidiodomycosis	0	0.00	0	0.00
Cryptococcosis	4	0.07	8	0.14
Cryptosporiodiosis	3	0.05	2	0.03
Herpes simplex	1	0.02	6	0.10
Herpes zoster – multidermatomal	1	0.02	2	0.03
Histoplasmosis	0	0.00	1	0.02
HIV Encephalopathy	1	0.02	0	0.00
Isosporiasis	1	0.02	0	0.00
Kaposis's sarcoma	2	0.03	2	0.03
Leishmaniasis – visceral	1	0.02	2	0.03
Lymphoma – Hodgkins	2	0.03	2	0.03
Lymphoma – non-Hodgkins	9	0.16	12	0.21
Lymphoma – primary brain	0	0.00	0	0.00
Microsporidiosis	0	0.00	0	0.00
Mycobacterium avium complex	0	0.00	0	0.00
Mycobacterial infection – other	0	0.00	1	0.02
Nocardiosis	0	0.00	0	0.00
Penicillium marneffei	0	0.00	0	0.00
Pneumocystis jiroveci pneumonia	6	0.10	7	0.12
Pneumocystis jiroveci extrapulmonary	0	0.00	0	0.00
PML	2	0.03	0	0.00
Rhodococcus equi disease	0	0.00	0	0.00
Salmonella septicemia, recurrent	0	0.00	0	0.00
Toxoplasmosis of the brain	2	0.03	1	0.02
Tuberculosis	8	0.14	3	0.05
Wasting syndrome	1	0.02	4	0.07

*Women only. Rates are per 100 person-years

Web Table IIb: Rate of Specific Opportunistic Diseases by Treatment Group - ESPRIT

Event	IL-2		Control	
	N	Rate	N	Rate
Aspergillosis	0	0.00	0	0.00
Bacterial pneumonia - recurrent	4	0.03	4	0.03
Bartonellosis	0	0.00	0	0.00
Candidiasis of the bronchi/trachea/lungs	0	0.00	0	0.00
Candidiasis esophageal	9	0.06	10	0.07
Cervical carcinoma*	4	0.15	4	0.15
Chagas disease of the CNS	0	0.00	0	0.00
CMV disease	5	0.04	0	0.00
Coccidiodomycosis	0	0.00	0	0.00
Cryptococcosis	3	0.02	1	0.01
Cryptosporiodiosis	4	0.03	1	0.01
Herpes simplex	1	0.01	1	0.01
Herpes zoster – multidermatomal	1	0.01	1	0.01
Histoplasmosis	1	0.01	0	0.00
HIV Encephalopathy	4	0.03	0	0.00
Isosporiasis	1	0.01	0	0.00
Kaposis's sarcoma	2	0.01	6	0.04
Leishmaniasis – visceral	0	0.00	1	0.01
Lymphoma – Hodgkins	5	0.04	7	0.05
Lymphoma – non-Hodgkins	12	0.08	10	0.07
Lymphoma – primary brain	1	0.01	1	0.01
Microsporidiosis	0	0.00	0	0.00
Mycobacterium avium complex	0	0.00	0	0.00
Mycobacterial infection – other	1	0.01	0	0.00
Nocardiosis	0	0.00	1	0.01
Penicillium marneffei	0	0.00	0	0.00
Pneumocystis jiroveci pneumonia	10	0.07	11	0.08
Pneumocystis jiroveci extrapulmonary	0	0.00	0	0.00
PML	1	0.01	0	0.00
Rhodococcus equi disease	0	0.00	0	0.00
Salmonella septicemia, recurrent	0	0.00	0	0.00
Toxoplasmosis of the brain	2	0.01	0	0.00
Tuberculosis	6	0.04	8	0.06
Wasting syndrome	2	0.01	2	0.01

*Women only. Rates are per 100 person-years

Web Table IIIa: Rate+ of Specific Causes of Death by Treatment Group - SILCAAT

	IL-2 Group		Control Group	
	N	Rate	N	Rate
Opportunistic Disease (OD)++	11	3.14	17	5.08
Cardiovascular disease or sudden death	7	2.00	13	3.89
Hepatic	9	2.57	5	1.49
Infection (excluding OD, hepatitis B and hepatitis C)	4	1.14	8	2.39
Non-AIDS malignancies	20	5.71	16	4.78
Accidents/violence	2	0.57	1	0.30
Substance abuse	0	0.00	2	0.60
Suicide	4	1.14	0	0.00
Other	11	3.14	3	0.90
Cause unknown	13	3.71	12	3.59

++ Deaths from OD included 3 IL-2 and 10 control deaths due to AIDS-malignancies.

+ Rates are per 100 person-years

Web Table IIIb: Rate[+] of Specific Causes of Death by Treatment Group - ESPRIT

	IL-2 Group		Control Group	
	N	Rate	N	Rate
Opportunistic Disease (OD)[++]	10	0.07	10	0.07
Cardiovascular disease or sudden death	11	0.08	16	0.11
Hepatic	8	0.06	14	0.10
Infection (excluding OD, hepatitis B and hepatitis C)	9	0.06	6	0.04
Non-AIDS malignancies	22	0.15	20	0.14
Accidents/violence	5	0.04	9	0.06
Substance abuse	5	0.04	8	0.06
Suicide	10	0.07	5	0.04
Other	13	0.09	11	0.08
Cause unknown	14	0.10	17	0.12

[++] Deaths from OD included 6 IL-2 and 10 control deaths due to AIDS-malignancies.

[+]Rates are per 100 person-years

Web Table IVa: Rate of Grade 4 Clinical Events by MedDRA Primary System Organ Class and Treatment Group - SILCAAT

System Organ Class	IL-2 No.	IL-2 Rate	Control No.	Control Rate
Blood and lymphatic system disorders	7	0.12	6	0.10
Cardiac disorders	27	0.47	29	0.51
Congenital, familial and genetic disorders	1	0.02	0	0.00
Ear and labyrinth disorders	0	0.00	0	0.00
Endocrine disorders	0	0.00	3	0.05
Eye disorders	0	0.00	1	0.02
Gastrointestinal disorders	44	0.78	25	0.44
General disorders and administration site conditions	11	0.19	5	0.09
Hepatobiliary disorders	24	0.42	18	0.31
Immune system disorders	1	0.02	2	0.03
Infections and infestations	50	0.89	51	0.90
Injury, poisoning and procedural complications	11	0.19	17	0.30
Investigations	1	0.02	2	0.03
Metabolism and nutrition disorders	6	0.10	6	0.10
Musculoskeletal and connective tissue disorders	10	0.17	11	0.19
Neoplasms benign, malignant and unspecified	41	0.72	35	0.62
Nervous system disorders	20	0.35	19	0.33
Pregnancy, puerperium, and perinatal conditions*	0	0.00	0	0.00
Psychiatric disorders	20	0.35	8	0.14
Renal and urinary disorders	11	0.19	18	0.32
Reproductive system and breast disorders	1	0.02	6	0.10
Respiratory, thoracic and mediastinal disorders	13	0.22	17	0.30
Skin and subcutaneous tissue disorders	3	0.05	2	0.03
Social circumstances	0	0.00	0	0.00
Surgical and medical procedures	3	0.05	1	0.02
Vascular disorders	10	0.17	7	0.12

* Women only. Rates are per 100 person-years

Web Table IVb: Rate of Grade 4 Clinical Events
by MedDRA Primary System Organ Class and Treatment Group - ESPRIT

System Organ Class	IL-2		Control	
	No.	Rate	No.	Rate
Blood and lymphatic system disorders	20	0.14	21	0.15
Cardiac disorders	58	0.42	53	0.39
Congenital, familial and genetic disorders	2	0.01	1	0.01
Ear and labyrinth disorders	3	0.02	0	0.00
Endocrine disorders	7	0.05	2	0.01
Eye disorders	8	0.06	7	0.05
Gastrointestinal disorders	66	0.47	63	0.46
General disorders and administration site conditions	41	0.29	21	0.15
Hepatobiliary disorders	32	0.23	25	0.18
Immune system disorders	6	0.04	3	0.02
Infections and infestations	94	0.68	89	0.66
Injury, poisoning and procedural complications	34	0.24	27	0.20
Investigations	22	0.16	12	0.09
Metabolism and nutrition disorders	22	0.16	14	0.10
Musculoskeletal and connective tissue disorders	21	0.15	26	0.19
Neoplasms benign, malignant and unspecified	61	0.43	60	0.44
Nervous system disorders	60	0.43	49	0.36
Pregnancy, puerperium, and perinatal conditions*	5	0.19	2	0.08
Psychiatric disorders	44	0.31	29	0.21
Renal and urinary disorders	26	0.18	26	0.19
Reproductive system and breast disorders	10	0.07	8	0.06
Respiratory, thoracic and mediastinal disorders	35	0.25	32	0.23
Skin and subcutaneous tissue disorders	8	0.06	6	0.04
Social circumstances	1	0.01	1	0.01
Surgical and medical procedures	2	0.01	3	0.02
Vascular disorders	40	0.28	14	0.10

Women only. Rates are per 100 person-years

The members of the ESPRIT and SILCAT study groups are as follows:

Coordinating Centers: Copenhagen: B Aagaard, E Aragon, J Arnaiz, L Borup, B Clotet, U Dragsted, A Fau, D Gey, J Grarup, U Hengge, P Herrero, P Jansson, B Jensen, K Jensen, H Juncher, P Lopez, J Lundgren, C Matthews, D Mollerup, M Pearson, A Phillips, S Reilev, K Tillmann, S Varea. **London:** B Angus, A Babiker, B Cordwell, J Darbyshire, W Dodds, S Fleck, J Horton, F Hudson, Y Moraes, F Pacciarini, A Palfreeman, N Paton, N Smith, F van Hooff. **Minneapolis:** J Bebchuk, G Collins, E Denning, A DuChene, L Fosdick, M Harrison, K Herman-Lamin, E Krum, G Larson, J Neaton, R Nelson, K Quan, S Quan, T Schultz, G Thompson, D Wentworth, N Wyman. **Sydney:** C Carey, F Chan, D Cooper, B Cordwell, D Courtney-Rodgers, F Drummond, S Emery, M Harrod, S Jacoby, L Kearney, M Law, E Lin, S Pett, R Robson, N Seneviratne, M Stewart, E Watts. **Washington:** E Finley, F Gordin, A Sánchez, B Standridge, M Vjecha.

Endpoint Review Committee: W Belloso, R Davey, D Duprez, J Gatell, J Hoy, A Lifson, C Pederson, G Perez, R Price, R Prineas, F Rhame, J Sampson, J Worley.

Data and Safety Monitoring Board: J Modlin, V Beral, R Chaisson, T Fleming, C Hill, K Kim, B Murray, B Pick, M Seligmann, I Weller.

National Institute of Allergy and Infectious Disease: K Cahill, L Fox, M Luzar, A Martinez, L McNay, J Pierson, J Tierney, S Vogel.

International Drug Distribution (CTS Inc., Durham, North Carolina): V Costas, J Eckstrand.

Specimen Repository (SAIC Frederick, Inc.*):* S Brown.

Clinical Sites for ESPRIT and/or SILCAAT: Argentina: L Abusamra, E Angel, S Aquilia, W Belloso, J Benetucci, V Bittar, E Bogdanowicz, P Cahn, A Casiro, J Contarelli, J Corral, L Daciuk, D David, W Dobrzanski, A Duran, J Ebenrstejin, I Ferrari, D Fridman, V Galache, G Guaragna, S Ivalo, A Krolewiecki, I Lanusse, H Laplume, M Lasala, R Lattes, J Lazovski, G Lopardo, M Losso, L Lourtau, S Lupo, A Maranzana, C Marson, L Massera, G Moscatello, S Olivia, I Otegui, L Palacios, A Parlante, H Salomon, M Sanchez, C Somenzini, C Suarez, M Tocci, J Toibaro, C Zala. **Australia:** S Agrawal, P Ambrose, C Anderson, J Anderson, D Baker, K Beileiter, K Blavius, M Bloch, M Boyle, D Bradford, P Britton, P Brown, T Busic, A Cain, L Carrall, S Carson, I Chenoweth, J Chuah, F Clark, J Clemons, K Clezy, D Cooper, P Cortissos, N Cunningham, M Curry, L Daly, C D'Arcy-Evans, R Del Rosario, S Dinning, P Dobson, W Donohue, N Doong, C Downs, E Edwards, S Edwards, C Egan, W Ferguson, R Finlayson, C Forsdyke, L Foy, T Franic, A Frater, M French, D Gleeson, J Gold, P Habel, K Haig, S

Hardy, R Holland, J Hoy, J Hudson, R Hutchison, N Hyland, R James, C Johnston, M Kelly, M King, K Kunkel, H Lau, J Leamy, D Lester, J Leung, A Lohmeyer, K Lowe, K MacRae, C Magness, O Martinez, H Maruszak, N Medland, S Miller, J Murray, P Negus, R Newman, M Ngieng, C Nowlan, J Oddy, N Orford, D Orth, J Patching, M Plummer, S Price, R Primrose, I Prone, H Ree, C Remington, R Richardson, S Robinson, G Rogers, J Roney, N Roth, D Russell, S Ryan, J Sarangapany, T Schmidt, K Schneider, C Shields, C Silberberg, D Shaw, J Skett, D Smith, T Meng Soo, D Sowden, A Street, B Kiem Tee, JI Thomson, S Topaz, R Vale, C Villella, A Walker, A Watson, N Wendt, L Williams, D Youds. **Austria:** A Aichelburg, P Cichon, B Gemeinhart, A Rieger, B Schmied, V Touzeau-Romer, N Vetter. **Belgium:** R Colebunders, N Clumeck, A DeRoo, K Kabeya, E O'Doherty, S de Wit. **Brazil:** C De Salles Amorim, C Basso, S Flint, E Kallas, G Levi, D Lewi, L Pereira Jr, M da Silva, T Souza, A Toscano. **Canada (CTN):** J Angel, M Arsenault, M Bast, B Beckthold, P Bouchard, I Chabot, R Clarke, J Cohen, P Coté, M Ellis, C Gagne, J Gill, M Houde, B Johnston, N Jubinville, C Kato, N Lamoureux, G Larson, J Latendre-Paquette, A Lindemulder, A McNeil, N McFarland, J Montaner, C Morrisseau, R O'Neill, G Page, A Piche, B Pongracz, H Preziosi, L Puri, A Rachlis, E Ralph, I Raymond, D Rouleau, JP Routy, R Sandre,T Seddon, S Shafran, C Sikora, F Smaill, D Stromberg, S Trottier, S Walmsley, K Weiss, K Williams, D Zarowny. **Denmark:** B Baadegaard, Å Bengaard Andersen, K Boedker, P Collins, J Gerstoft, L Jensen, H Moller, P Lehm Andersen, I Loftheim, L Mathiesen, H Nielsen, N Obel, C Pedersen, D Petersen, L Pors Jensen, F Trunk Black. **France (ANRS):** JP Aboulker, A Aouba, M Bensalem, H Berthe, C Blanc, M Bloch, D Bornarel, O Bouchaud, F Boue, E Bouvet, C Brancon, S Breaud, D Brosseau, A Brunet, C Capitant, C Ceppi, C Chakvetadze, C Cheneau, JM Chennebault, P De Truchis, AM Delavalle, JF Delfraissy, P Dellamonica, AM Delavalle, C Dumont, N Edeb, G Fabre, S Ferrando, A Foltzer, V Foubert, JA Gastaut, J Gerbe, PM Girard, C Goujard, B Hoen, P Honore, H Hue,T Hynh, C Jung, S Kahi, C Katlama, JM Lang, V Le Baut, B Lefebvre, N Leturque, Y Lévy, J Loison, G Maddi, A Maignan, C Majerholc, C de Boever, JL Meynard, C Michelet, C Michon, M Mole, E Netzer, G Pialoux, I Poizot-Martin, F Raffi, M Ratajczak, I Ravaux, J Reynes, D Salmon-Ceron, M Sebire, A Simon, L Tegna, D Tisne-Dessus, C Tramoni, JP Viard, M Vidal, C Viet-Peaucelle, L Weiss, A Zeng, D Zucman. **Germany:** A Adam, K Arastéh, G Behrens, F Bergmann, M Bickel, D Bittner, J Bogner, N Brockmeyer, N Darrelmann, M Deja, M Doerler, S Esser, G Faetkenheuer, S Fenske, S Gajetzki, D Gey, F Goebel, D Gorriahn, E Harrer, T Harrer, H Hartl, M Hartmann, S Heesch, W Jakob, H Jäger, H Klinker, G Kremer, C Ludwig, K Mantzsch, S Mauss, A Meurer, A Niedermeier, N Pittack, A Plettenberg, A Potthoff, M Probst, M Rittweger, J Rockstroh, B Ross, J Rotty, E Rund, T Ruzicka, Rt Schmidt, G Schmutz, E Schnaitmann, D Schuster, T Sehr, B Spaeth, S Staszewski, HJ Stellbrink, C Stephan, T Stockey, A Stoehr, K Tillmann, A Trein, T Vaeth, M Vogel, J Wasmuth, C Wengenroth, R Winzer, E Wolf. **Ireland:** F Mulcahy, DI Reidy. **Israel:** Y Cohen, G Drora, I Eliezer, O Godo, E Kedem, E Magen, M Mamorsky, S Pollack, Z Sthoeger, H Vered, I Yust. **Italy:** F Aiuti, M Bechi, A Bergamasco, D Bertelli, R Bruno, L Butini, M Cagliuso,

G Carosi, S Casari, V Chrysoula, G Cologni, V Conti, A Costantini, A Corpolongo, G D'Offizi, F Gaiottino, M Di Pietro, R Esposito, G Filice, M Francesco, E Gianelli, C Graziella, L Magenta, F Martellotta, R Maserati, F Mazzotta, G Murdaca, G Nardini, S Nozza, F Puppo, M Pogliaghi, D Ripamonti, C Ronchetti, S Rusconi, V Rusconi, P Sacchi, N Silvia, F Suter, G Tambussi, A Uglietti, M Vechi, B Vergani, F Vichi, P Vitiello. **Japan:** A Iwamoto, Y Kikuchi, N Miyazaki, M Mori, T Nakamura, T Odawara, S Oka, T Shirasaka, M Tabata, M Takano, C Ueta, D Watanabe, Y Yamamoto. **Morocco:** I Erradey, H Himmich, K Marhoum El Filali. **The Netherlands:** W Blok, R van Boxtel, K Brinkman H Doevelaar, A van Eeden, M Grijsen, M Groot, J Juttmann, M Kuipers, S Ligthart, P van der Meulen, J Lange, N Langebeek, S Ligthart, P Reiss, C Richter, M Schoemaker, L Schrijnders-Gudde, E Septer-Bijleveld, H Sprenger, J Vermeulen, R ten Kate, B van de Ven**.** **Norway:** J Bruun, D Kvale, A Maeland. **Poland:** E Bakowska, M Beniowski, A Boron-Kaczmarska, J Gasiorowski, A Horban, M Inglot, B Knysz, E Mularska, M Parczewski, M Pynka, W Rymer, A Szymczak. **Portugal:** M Aldir, F Antunes, C Baptista, J da Conceicao Vera, M Doroana, K Mansinho, C Raquel A dos Santos, E Valadas, I Vaz Pinto. **Singapore:** E Chia, E Foo, F Karim, PL Lim, A Panchalingam, N Paton, A Quek. **Spain:** R Alcázar-Caballero, E Aragon, J Arnaiz, J Arribas, J Arrizabalaga, X de Barron, F Blanco, E Bouza, I Bravo, S Calvo, L Carbonero, I Carpena, M Castro, B Clotet, L Cortes, M del Toro, P Domingo, M Elias, J Espinosa, V Estrada, E Fernandez-Cruz, P Fernández, H Freud, M Fuster, A Garcia, G Garcia, R Garrido, J Gatell, P Gijón, J Gonzalez-García, I Gil, A González, J González-Lahoz, P López Grosso, M Gutierrez, E Guzmán, J Iribarren, M Jiménez, A Jou, J Juega, J Lopez, P Lopez, F Lozano, L Martín-Carbonero, R Mata, G Mateo, A Menasalvas, C Mirelles, J de Miguel Prieto, M Montes, A Moreno, J Moreno, V Moreno, R Muñoz, A Ocampo, E Ortega, L Ortiz, B Padilla, A Parras, A Paster, J Pedreira, J Peña, R Perea, B Portas, J Puig, F Pulido, M Rebollar, J de Rivera, V Roca, F Rodríguez- Arrondo, R Rubio, J Santos, J Sanz, G Sebastian, M Segovia, V Soriano, L Tamargo, S Varea, P Viciana, M von Wichmann. **Sweden:** G Bratt, A Hollander, P Olov Pehrson, I Petz, E Sandstrom, A Sönnerborg. **Switzerland:** E Bernasconi, V Gurtner. **Thailand:** U Ampunpong, C Auchieng, C Bowonwatanuwong, P Chanchai, P Chetchotisakd, T Chuenyan, C Duncombe, M Horsakulthai, P Kantipong, K Laohajinda, P Phanuphak, V Pongsurachet, S Pradapmook, K Ruxruntham, S Seekaew, A Sonjai, S Suwanagool, W Techasathit, S Ubolyam, J Wankoon. **United Kingdom:** I Alexander, D Dockrell, P Easterbrook, B Edwards, E Evans, M Fisher, R Fox, B Gazzard, G Gilleran, J Hand, L Heald, C Higgs, S Jebakumar, I Jendrulek, M Johnson, S Johnson, F Karim, G Kinghorn, K Kuldanek, C Leen, R Maw, S McKernan, L McLean, S Morris, M Murphy, S O'Farrell, E Ong, B Peters, C Stroud, M Wansbrough-Jones, J Weber, D White, I Williams, M Wiselka, T Yee. **United States:** S Adams, D Allegra, L Andrews, B Aneja, G Anstead, R Arduino, R Artz, J Bailowitz, S Banks, J Baxter, J Baum, D Benator, D Black, D Boh, T Bonam, M Brito, J Brockelman, S Brown, V Bruzzese, A Burnside Jr., V Cafaro, K Casey, L Cason, G Childress, CI Clark, D Clifford, M Climo, D Cohn, P Couey, H Cuervo, R Davey Jr, S Deeks, M Dennis, M Diaz-Linares, D Dickerson, M Diez, J Di Puppo, P Dodson, D Dupre, R Elion,

K Elliott, W El-Sadr, M Estes, J Fabre, M Farrough, J Flamm, S Follansbee, C Foster, C Frank, J Franz, G Frechette, G Freidland, J Frische, L Fuentes, C Funk, C Geisler, K Genther, M Giles, M Goetz, M Gonzalez, C Graeber, F Graziano, D Grice, B Hahn, C Hamilton, S Hassler, A Henson, S Hopper, M John, L Johnson, M Johnson, R Johnson, R Jones, J Kahn, M Kelly, N Klimas, M Kolber, S Koletar, A Labriola, R Larsen, F Lasseter, M Lederman, T Ling, T Lusch, R MacArthur, C Machado, L Makohon, J Mandelke, S Mannheimer, N Markowitz, M Martínez, N Martinez, M Mass, H Masur, D McGregor, D McIntyre, J McKee, D McMullen, M Mettinger, S Middleton, J Mieras, D Mildvan, P Miller, T Miller, V Mitchell, R Mitsuyasu, A Moanna, C Mogridge, F Moran, R Murphy, D Mushatt, R Nahass, D Nixon, S O'Brien, J Ojeda, P Okhuysen, M Olson, J Osterberger, W Owen, Sr. S Pablovich, S Patel, G Perez, G Pierone Jr., R Poblete, A Potter, E Preston, C Rappoport, N Regevik, M Reyelt, F. Rhame, L Riney, M Rodriguez-Barradas, M Rodriguez, Milagros Rodriguez, J Rodriguez, R Roland, C Rosmarin-DeStefano, W Rossen, J Rouff, M Saag, J Sampson, S Santiago, J Sarria, S Wirtz, U Schmidt, C Scott, A Sheridan, A Shin, S Shrader, G Simon, D Slowinski, K Smith, J Spotkov, C Sprague, D States, C Suh, J Sullivan, K Summers, B Sweeton, V Tan, T Tanner, E Tedaldi, Z Temesgen, D Thomas, M Thompson, C Tobin, N Toro, W Towner, K Upton, J Uy, S Valenti, C van der Horst, J Vita, J Voell, J Walker, T Walton, K Wason, V Watson, A Wellons, J Weise, M White, T Whitman, B Williams, N Williams, J Windham, M Witt, K Workowski, G Wortmann, T Wright, C Zelasky, B Zwickl.
